# Rare distribution of butenolide-type signaling molecules among *Streptomyces* strains and functional importance as inducing factors for secondary metabolite production in *Streptomyces rochei* 7434AN4

**DOI:** 10.1038/s41429-025-00840-9

**Published:** 2025-06-12

**Authors:** Asahi Hirata, Miho Sumiyoshi, Hazuki Fujita, Momoko Akimoto, Mary Hannah Rose A. Padayao, Yuto Eguchi, Maki Matsuura, Miyuki Otsuka, Kuninobu Inada, Aiko Teshima, Kenji Arakawa

**Affiliations:** 1 Program of Biotechnology, Graduate School of Integrated Sciences for Life, Hiroshima University, Hiroshima, Japan; 2 Hiroshima Research Center for Healthy Aging (HiHA), Hiroshima University, Hiroshima, Japan; 3 Department of Molecular Biotechnology, Graduate School of Advanced Sciences of Matter, Hiroshima University, Hiroshima, Japan; 4 Faculty of Engineering, Hiroshima University, Hiroshima, Japan; 5 College of Agriculture, Tamagawa University, Machida, Tokyo Japan; 6 Natural Science Center for Basic Research and Development, Hiroshima University, Hiroshima, Japan

**Keywords:** Multienzyme complexes, Biocatalysis

## Abstract

*Streptomyces rochei* 7434AN4 produces two structurally unrelated polyketide antibiotics, lankacidin (LC) and lankamycin (LM), and their biosynthesis is tightly controlled by 2,3-disubstituted butenolide-type signaling molecules SRB1 and SRB2. We here investigated the distribution of 2,3-disubstituted butenolides (SRB-type butenolides) among randomly selected 122 *Streptomyces* strains using two approaches; (1) feeding of their culture extracts into an *srrX*-deficient strain KA20 of *S. rochei*, and (2) co-fermentation with strain KA20. All the randomly selected donor strains, except for *Streptomyces cellostaticus* (a LC and LM producer), failed to restore LC and LM production in strain KA20. These findings strongly revealed the rare distribution of SRB-type butenolide molecules in *Streptomyces* species. One of the SRB-type butenolide, SAB1, an inducing molecule for nikkomycin production in *Streptomyces ansochromogenes*, was unable to restore antibiotic production in strain KA20 even at 1 mM concentration. Furthermore, we noticed the accumulation of 4-dehydroxy-SRB1 as a novel compound when SRB1 was fed into strain KA20. Purified 4-dehydroxy-SRB1 has no inducing activity of antibiotic production in strain KA20 even at 1000-fold higher concentration (50 µM) against a minimum inducing concentration of natural SRB1 (40 nM). These findings suggested the importance of the length of a hydrocarbon chain attached at C-2 and a hydroxyl group at C-4 for inducing activity in *S. rochei*.

## Introduction

**Fig. 1:**
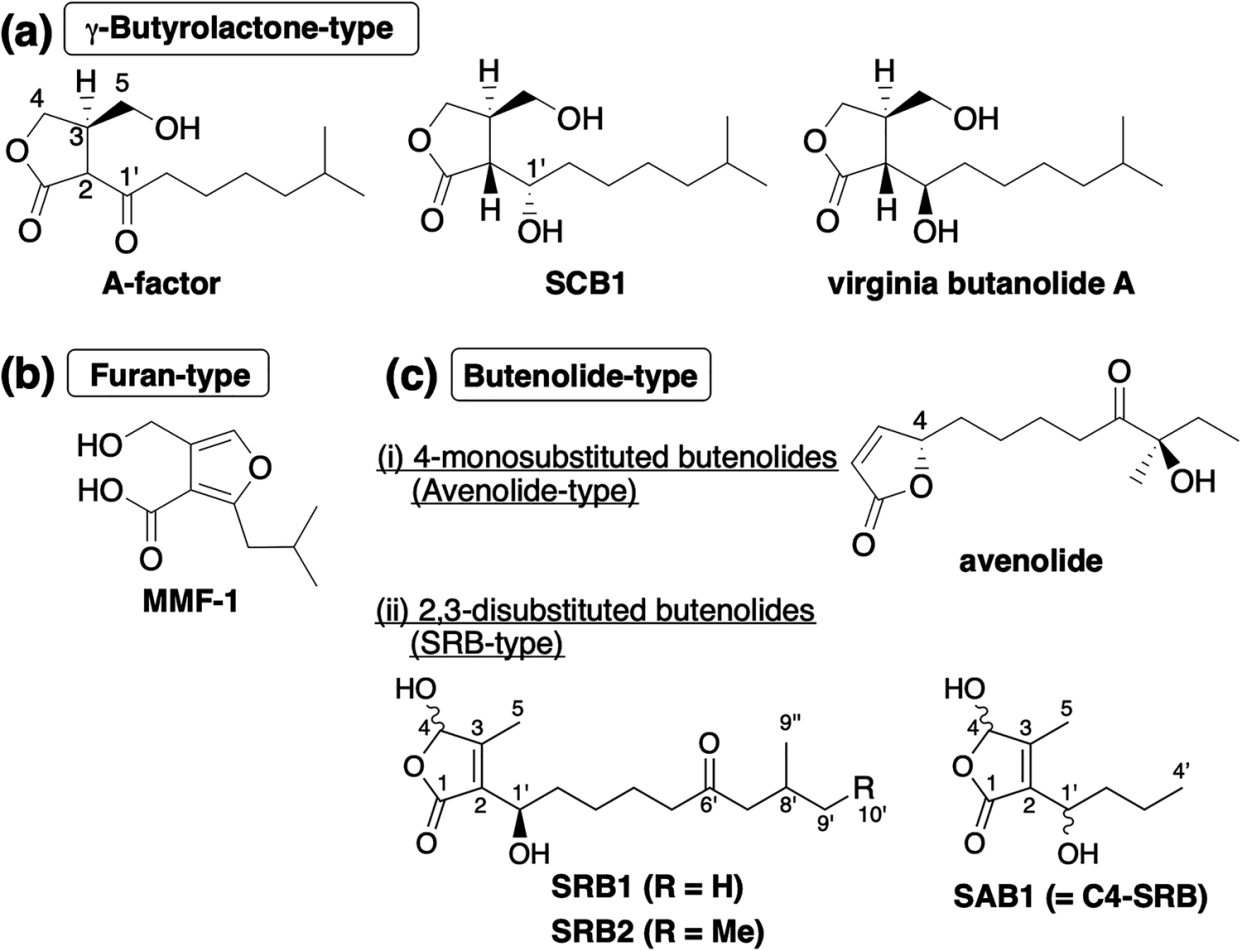
Representative *Streptomyces* signaling molecules. **a** γ-Butyrolactone-type molecules. A-factor from *Streptomyces griseus*, SCB1 from *Streptomyces coelicolor*, and virginia butanolide A from *Streptomyces virginiae*. **b** Furan-type molecules. Methylenomycin furan MMF-1 from *Streptomyces coelicolor*. **c** (i) Avenolide-type butanolide. Avenolide from *Streptomyces avermectinius*. (ii) SRB-type butenolides. Natural signaling molecules SRB1 and SRB2 isolated from *S. rochei* 7434AN4, and an SRB analog, SAB1, isolated from *Streptomyces ansochromogenes*. Other signaling molecules are shown in Fig. S1

The filamentous soil bacterial genus, *Streptomyces*, is characterized by their distinct properties to produce a variety of secondary metabolites, including antibiotics, antitumor agents, immunosuppressants, herbicides, and insecticides. In general, secondary metabolite production is tightly controlled by small diffusible signaling molecules [1, 2]. The *Streptomyces* signaling molecules hitherto identified are classified into three groups; γ-butyrolactones, furans, and butenolides (Fig. 1 and S1) [3–7]. For example, γ-butyrolactone group includes A-factor in *Streptomyces griseus* for streptomycin production [8], SCBs in *Streptomyces coelicolor* A3(2) [9–12], and virginia butanolides in *Streptomyces virginiae* (Fig. 1a and S1a) [13]. Furan group includes methylenomycin furans in *Streptomyces coelicolor* for methylenomycin production (Fig. 1b and S1b) [14]. Then, butenolide group is further classified into two subgroups; (i) 4-monosubstituted butenolides including avenolide in *Streptomyces avermitilis* (synonym: *Streptomyces avermectinius*) for avermectin (Fig. 1c-i and S1c-i) [15], and (ii) 2,3-disubstituted butenoliodes including SRBs in *Streptomyces rochei* 7434AN4 for lankacidin (LC) and lankamycin (LM) [16, 17] and SABs in *Streptomyces ansochromogenes* for nikkomycin (Fig. 1c-ii and S1c-ii) [18].

*Streptomyces rochei* strain 7434AN4 produces two structurally unrelated polyketide antibiotics, LC and LM (Fig. S2), and harbors three linear plasmids, pSLA2-L, -M, and -S [19, 20]. LC and LM inhibit peptide synthesis synergistically by binding to neighboring sites in bacterial ribosome [21, 22]. The considerable antitumor activity of lankacidins is associated with a paclitaxel-like mode of action [23–26]. Their biosynthetic genes together with regulatory genes are coded on a giant linear plasmid pSLA2-L (210,614 bp) [27–33]. Our group has revealed that an SRB biosynthesis gene (*srrX*), an SRB receptor gene (*srrA*), and two SARP (*Streptomyces* antibiotic regulatory protein) genes (*srrY* and *srrZ*) constitute the regulatory pathway for LC and LM production in *S. rochei* 7434AN4. This regulatory pathway goes from *srrX* through *srrA* to *srrY*, leading to LC production [34, 35], while, for LM production, *srrY* directly activates the transcription of *srrZ* (Fig. S2) [36]. Furthermore, the chemical structures of signaling molecules SRB1 and SRB2 in *S. rochei* were determined to be 2-(1’-hydroxyl-6’-oxo-8’-methylnonyl)-3-methyl-4-hydroxybut-2-en-1,4-olide (for SRB1) and 2-(1’-hydroxyl-6’-oxo-8’-methyldecyl)-3-methyl-4-hydroxybut-2-en-1,4-olide (for SRB2) (Fig. 1c-ii and S1c-ii) [16]. They are first examples of 2,3-disubstituted butenolides (termed as SRB-type butenolides) that could act as inducers for antibiotic production in *Streptomyces*. Their C1’ stereochemistry was determined to be *R* based on chiral HPLC analysis and chemical synthesis [16], and then we elucidated a final biosynthetic step for SRBs, in which a P450 monooxygenase gene *srrO* is responsible for an oxidation step to introduce C-6’ ketone group [17]. Possible biosynthetic pathway for SRBs was shown in Fig. S3. Analogously to the biosynthesis of γ-butyrolactone molecules including A-factor, SrrX protein is responsible for condensation between a C3 unit and C12 or C13 β-keto thioester derivatives, the resultant of which then receives intramolecular aldol condensation to form a butenolide skeleton. This may be a common precursor for SRB-type butanolide and γ-butyrolactone molecules. In the former case, butenolide may be further modified by several enzymes including a phosphatase protein SrrP, a dehydrogenase protein SrrG, and a P450 monooxygenase protein SrrG. On the other hand, in the latter case, enoyl reduction occurs at C-2,3 position, and then C-1’ carbonyl, in some cases, accepts stereospecific reduction for the biosynthesis of γ-butyrolactone. Thus, butenolide signaling molecules are worth studying in view of biosynthesis and inducing activity.

Regarding to structural diversity, about 60% of *Streptomyces* strains were estimated to produce γ-butyrolactone-type molecules for induction of antibiotic production [8, 37]. Furthermore, avenolide-producing activity was detected in 24% of actinomycetes [38]. Distribution of other-type signaling molecules, including SRB-type butenolides, furan-type, and uncharacterized novel-type molecules is worth studying in view of their structural basis of regulatory pathway in *Streptomyces* species and further application for genome mining. Remarkably, the other SRB-type molecules termed as SABs (Fig. 1c-ii and S1c-ii) were isolated as inducing elements for nikkomycin production in *Streptomyces ansochromogenes* [18], whose differences against SRBs are the length of hydrocarbon chains attached at C-2 position of butenolides, indicating the structural diversity of SRB-type butenolides in *Streptomyces* species.

We here investigated the structural diversity of SRB-type butenolides in *Streptomyces* species using randomly selected 122 strains, the results of which were described in this paper.

## Materials and methods

### Strains, reagents, and culture conditions

All *Streptomyces* strains used in this study were listed in Table S1. *S. rochei* strain 51252 that harbors a giant linear plasmid pSLA2-L was used as a parent strain [19, 27]. Strain KA20, a double mutant of *srrX* and the transcriptional repressor gene *srrB* in strain 51252, was used as an SRB indicator strain [34, 39]. YM medium (0.4% yeast extract, 1.0% malt extract, and 0.4% D-glucose, pH 7.3) was used for secondary metabolite production and bioassay.

### Spectroscopic instruments

NMR spectra were recorded on JEOL ECA-500 and/or ECA-600 spectrometers equipped with a field gradient accessory. Deuteriochloroform (99.8 atom% enriched; Kanto Chemical Co., Ltd., Tokyo, Japan) was used as a solvent. Chemical shifts were recorded as a *δ* value based on a resident solvent signal (*δ*_C_ = 77.0), or an internal standard signal of tetramethylsilane (*δ*_H_ = 0). The coupling constants were recorded as a *J* value in Hz. High resolution electrospray ionization (ESI) mass spectra were measured by a LTQ Orbitrap XL mass spectrometer (Thermo Fisher Scientific, Waltham, MA, USA).

### Evaluation of structural diversity of SRB in the *Streptomyces* culture library

#### Feeding approach

A total of 122 *Streptomyces* strains (donors) were cultured in YM medium (100 mL) in a 500-mL Sakaguchi flask at 28 °C for 3 days, and then each culture broth was extracted by equal volume of EtOAc twice. The combined organic phase was dried (Na_2_SO_4_), filtered, and concentrated to dryness. The resulting culture extracts through EtOAc-extraction were dissolved in MeOH (500 µL), and then an aliquot (50 µL) was fed into strain KA20 in 5 mL of YM liquid medium. Two-days culture was extracted with EtOAc, and the organic phase was dried (Na_2_SO_4_), filtered, and concentrated to dryness. Restoration of LC and LM production in strain KA20 was analyzed by bioassay using *Micrococcus luteus*, high performance liquid chromatography (HPLC), and thin layer chromatography (TLC). Bioassay was performed according to our previous literature [29]. The crude extract (50 µL) dissolved in 500-µL MeOH was spotted onto disc filter paper (8 mm diameter), and the YM agar plates overlaid with *M. luteus* were incubated at 28 °C for 1 day. For HPLC analysis, the crude extract was applied on a COSMOSIL CHOLESTER column (4.6 I.D. x 250 mm, Nacalai Tesque, Kyoto, Japan) and eluted with a mixture of acetonitrile-10 mM sodium phosphate buffer (pH 8.2) (3:7, *v*/*v*) at a flow rate of 1.0 mL min^–1^. The eluate was monitored at 230 nm with an MD-2010 multiwavelength photodiode array detector (JASCO Corporation, Tokyo, Japan). TLC was developed with a mixture of CHCl_3_–MeOH (15:1, *v*/*v*) and baked after spraying with anisaldehyde-H_2_SO_4_.

To investigate whether *S. cellostaticus*, known as producer for LC and LM, could accumulate SRBs, its one-day-growth culture extract (4 L) was partially purified by Sephadex LH20 chromatography with MeOH as eluent. Then, aliquots of fractions (10 µL) were fed into strain KA20 (5 mL), and the resulting cultures grown for 2 days were analyzed by TLC.

#### Co-fermentation approach

All donor strains were separately co-cultured with strain KA20 in YM medium (100 mL) in a 500-mL Sakaguchi flask at 28 °C for 3 days. As for comparison, all the donor strains were independently cultured in same condition. The culture supernatants were extracted with equal volume of EtOAc twice, and the combined organic phase was dried with Na_2_SO_4_, filtered, and concentrated to dryness. The culture extracts were dissolved in MeOH (500 µL), and each of their aliquots (50 µL) was subjected to bioassay using *Micrococcus luteus* as an indicator microorganism. Increase of the inhibitory zone in co-cultured samples indicated the induction of antibiotic production in strain KA20 by diffusible element(s) such as signaling molecule(s) from the donor strains.

### Synthesis of 2-(1’-hydroxybutyl)-3-methyl-4-hydroxybut-2-en-1,4-olide SAB1 ( = C4-SRB)

A solution of *n*-butyl lithium (1.40 mL, 1.64 M in hexane, 2.31 mmol) was dropwisely added to a solution of diisopropylamine (320 µL, 2.28 mmol) in THF (8 mL) at –78 °C. After 30 min of stirring, hexamethylphosphoric triamide (HMPA) (2.50 mL) was added to the mixture at –78 °C. A solution of 3-methyl-4-(L-menthyloxy)but-2-en-1,4-olide (550 mg, 2.18 mmol) [16, 17, 40, 41] in THF (8 mL) was added to the mixture at –78 °C over 15 min, and the mixture was further stirred at the same temperature for 1 h. Then, a solution of 1-butanal (550 mg, 2.18 mmol) in THF (8 mL) was added dropwise at –78 °C over 10 min, and the mixture was further stirred at the same temperature for 2 h. Saturated aqueous NH_4_Cl (20 mL) was added to the mixture, and the resultant was extracted with CH_2_Cl_2_ twice. The combined organic phases were washed with brine, dried (Na_2_SO_4_), filtered, and concentrated to dryness. The residue was purified by silica gel chromatography with hexane–EtOAc (7:1–4:1, *v*/*v*) to give a 1:1 mixture of menthyl butenolide with a butyryl side chain (148 mg, 21%) as a colorless oil.

A solution of menthyl butenolide (120 mg, 370 µmol) above mentioned in CH_2_Cl_2_ (10 mL) was treated with 15% BBr_3_ solution in CH_2_Cl_2_ (2.00 mL, 3.15 mmol) at –78 °C, and the mixture was stirred at –78 °C for 1 h. Saturated aqueous NaHCO_3_ (5 mL) was carefully added, and the mixture was extracted with EtOAc twice. The combined organic phases were washed with brine, dried (Na_2_SO_4_), filtered, and concentrated to dryness. The residue was purified by silica gel chromatography with hexane-EtOAc (1:1, *v*/*v*) to give SAB1 ( = C4-SRB) (58 mg, 75%) as a colorless oil.

Mixture of C-4 epimers and C-1’ diastereomers; ^1^H NMR (CDCl_3_): *δ* = 0.94 (t, *J* = 7.5 Hz, H-4’, 3H), 1.32 (m, H-3’a, 1H), 1.42 (m, H-3’b, 1H), 1.64 (m, H-2’a, 1H), 1.81 (m, H-2’b, 1H), 2.07 (s, H-5, 3H), 4.49 (m, H-1’, 1H), 5.86 (brs, H-4, 1H); ^13^C NMR (CDCl_3_): *δ* = 11.4/11.6 (C-5), 13.7 (C-4’), 18.7 (C-3’), 37.8/38.3 (C-2’), 66.4 (C-1’), 98.8/98.9 (C-4), 129.9/130.4 (C-2), 157.9 (C-3), 171.7/172.0 (C-1); High resolution ESI-MS: observed *m*/*z* 209.0781 [M+Na]^+^ (calcd for C_9_H_14_O_4_Na, 209.0784). Spectral data was shown in Figs. S7–S12.

### In vivo synthesis of (1’*R*)-4-dehydroxy SRB1

Methanol solution of synthetic (1’*R*)-SRB1 (16 mg in 2-mL of MeOH) [16] was fed into strain KA20 in 2 L of YM liquid medium. Two-days culture was extracted with EtOAc, and the organic phase was dried (Na_2_SO_4_), filtered, and concentrated to dryness. The residue contains new greenish spot at Rf = 0.75, together with LC (Rf = 0.40) and LM (Rf = 0.65) on TLC developed with CHCl_3_–MeOH = 15:1 (*v*/*v*) and baked after spraying with anisaldehyde. The residue was purified by silica gel chromatography with CHCl_3_–MeOH (50:1 ~ 10:1, *v*/*v*) to give (1’*R*)-4-dehydroxy SRB1 (4.8 mg) as a colorless oil.

^1^H NMR (CDCl_3_): *δ* = 0.91 (d, *J* = 6.6 Hz, H-9’ and 9”, 6H), 1.31 and 1.31 (m, H-3’a, 1H), 1.41 (m, H-3’b, 1H), 1.60 (m, H-4’a, 2H), 1.66 (m, H-2’a, 1H), 1.86 (m, H-2’b, 1H), 2.08 (s, H-5, 3H), 2.13 (m, H-8’, 1H), 2.27 (d, *J* = 7.2 Hz, H-7’, 1H), 2.40 (t, *J* = 7.2 Hz, H-5’, 2H), 4.50 (t, *J* = 7.2 Hz, H-1’, 1H), 4.66 (d, *J* = 7.2 Hz, H-4, 2H); ^13^C NMR (CDCl_3_): *δ* = 12.2 (C-5), 22.6 (C-9’ and 9”), 23.2 (C-4’), 24.6 (C-8’), 25.1 (C-3’), 36.2 (C-2’), 43.0 (C-5’), 51.9 (C-7’), 66.6 (C-1’), 72.7 (C-4), 128.1 (C-2), 157.5 (C-3), 174.0 (C-1), 210.9 (C-6’); High resolution ESI-MS: observed *m*/*z* 291.1565 [M+Na]^+^ (calcd for C_15_H_24_O_4_Na, 291.1567). NMR spectral data was shown in Figs. S13–S18.

### Antibiotic-inducing activity of SRB derivatives in *S. rochei*

Antibiotic-inducing activity of two SRB derivatives, SAB1 and 4-dehydroxy-SRB1, were evaluated according to the procedure described in above section.

## Results and discussion

### In silico analysis of SRB biosynthetic protein SrrX

**Fig. 2:**
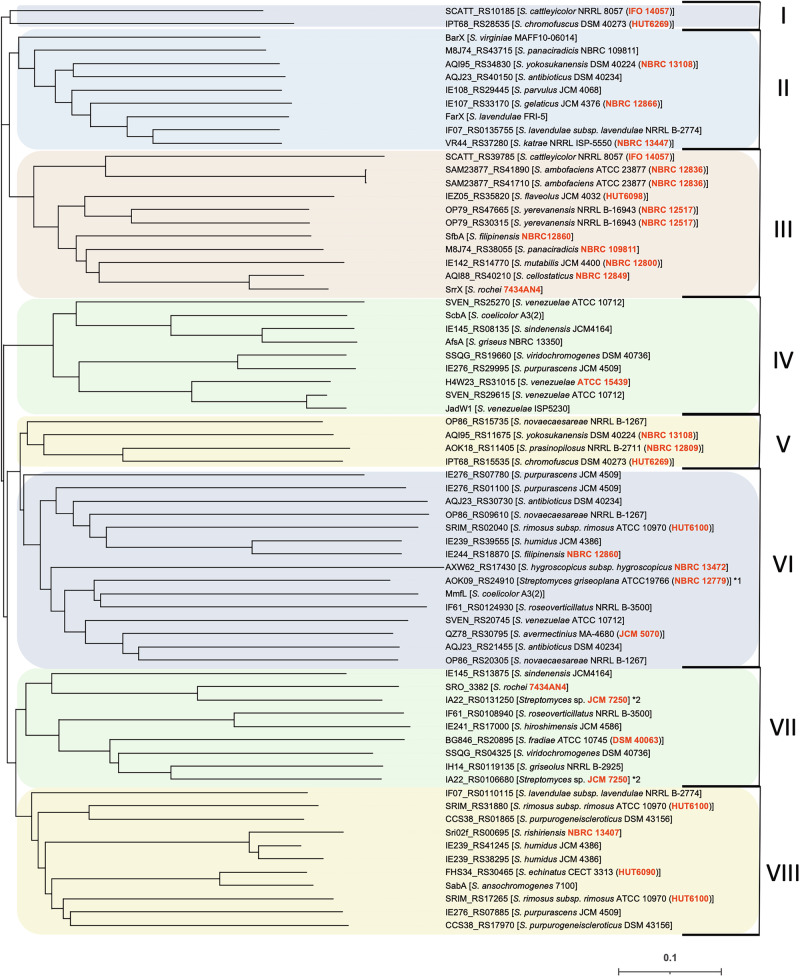
Phylogenetic tree of the SrrX (AfsA) homologous proteins in 70 *Streptomyces* species. Phylogenetic analysis was performed by Clustal omega software at Job Dispatcher site (https://www.ebi.ac.uk/jdispatcher/), and the rooted tree was drawn using iTOL (Interactive Tree of Life) web service (https://itol.embl.de). Red bold letters indicate 23 *Streptomyces* strains that were subjected to both feeding and co-fermentation experiments in this study. Scale bar: 0.1 changes per amino acid position. The representative SrrX homologs with relative amino-acid similarity and identity against SrrX were below mentioned: AfsA (37% identity, 75% similarity), ScbA (37%, 79%), JadW1 (38%, 80%), BarX (44%, 80%), FarX (42%, 78%), MmfL (30%, 71%), SabA (42%, 77%), SfbA (55%, 84%), M8J74_RS38055 (55%, 86%), and AQI88_RS40210 (86%, 96%). *1; This strain is proposed as *Streptacidiphilus griseoplanus*. *2; This strain is proposed as *Kitasatosporia papulose*

We performed in silico analysis of the signaling molecule biosynthetic enzymes in *Streptomyces* species. Among the *Streptomyces* signaling molecules hitherto identified, γ-butyrolactones, furans, and SRB-type butenolides are synthesized by AfsA (SrrX) homologous proteins, whereas an acyl-CoA oxidase homolog is responsible for the synthesis of avenolide. To investigate the distribution of SRB-type butenolides in terms of their synthetic enzymes, 70 of AfsA (SrrX) homologs deposited in GenBank were aligned and their phylogenetic tree was constructed (Fig. 2). They were classified into 8 clades, among which SrrX and its higher homologs were located in Clade III. Clade III includes AQI88_RS40210 protein from *Streptomyces cellostaticus* NBRC12849 (86% identity and 96% similarity to SrrX), SfbA protein from *Streptomyces filipinensis* NBRC12860 (55%, 84%), and M8J74_RS38055 protein from *Streptomyces panaciradicis* NBRC109811 (55%, 86%). *S. cellostaticus* produces LC and LM in a comparable yield to *S. rochei* 7434AN4. Other Clades contain six biosynthetic enzymes for hitherto identified signaling molecules (Fig. 1 and S1), including five γ-butyrolactones synthetic enzymes (ScbA, AfsA, JadW1, FarX, and BarX) and one furan-type molecule biosynthetic enzyme (MmfL), whose amino-acid identities against SrrX were below 44%. Remarkably, SabA protein from *S. ansochromogene*s is located in Clade VIII and has only 42% identity (122 aa in 285 aa) and 77% similarity (222 aa in 285 aa) to SrrX protein, although both of them produce SRB-type butenolides.

In general, the amino acid sequences of AfsA (SrrX) homologs have relatively lower identity (below 50%) even they produce same (or similar) signaling molecules. For the instance, ScbA protein in *S. coelicolor* and JadW1 protein in *S. venezuelae* are able to produce same signaling molecule termed as SCB3 (for *S. coelicolor*) and SVB1 (for *S. venezuelae*) (Fig. S1) [42], however, their amino acid sequence homologies are only 45% identity (129 aa in 285 aa) and 83% similarity (237 aa in 285 aa). Thus, it is difficult to predict the type of signaling molecules in a view of the sequence homology in biosynthetic enzymes.

### Structural diversity of SRB-type butenolides in *Streptomyces* species

The presence of SRB-type butenolides in randomly selected 122 *Streptomyces* strains (Table S1) was investigated through the restoration activity of LC and LM production in *S. rochei* strain KA20, an *srrX*-deficient mutant constructed previously [34]. Screening of SRB-type molecules was performed by two approaches; (1) feeding of their culture extracts into strain KA20, and (2) co-fermentation with an *srrX*-deficient strain KA20 of *S. rochei*.

In a feeding approach, the culture extracts from 122 donor strains (50 µL from 500 µL methanol solution prepared from 100-mL culture; equivalent to 10-mL culture) were separately added to the 5-mL culture of strain KA20, and then the restoration activity of LC and LM production was examined by bioassay using *Micrococcus luteus*. The culture extract of strain KA61 was used as a positive control for the source of SRBs in *S. rochei*. This strain has a mutation on an SARP-type activator gene *srrY*, which in turn blocks the downstream regulatory pathway for LC and LM production in *S. rochei* (Fig. S2) [35]. As a positive control, the feeding of the culture extract of *S. rochei* strain KA61 into strain KA20 showed considerable inhibitory zone on *M. luteus*, indicating the transfer of SRB molecules from strain KA61 into strain KA20 to result in LC and LM production (Tables S1 and S2). Hence, 122 culture extracts were separately fed into strain KA20, and their supernatants were extracted and put onto disk filters to judge the production of antibiotics in KA20. As shown in Table S1, 17 culture extracts led to growth inhibition of strain KA20, which was possibly due to the presence of growth inhibitory molecule(s) such as antibiotics being sensitive against *S. rochei*. Eighteen fed cultures showed considerable inhibitory zone against *M. luteus* (Table S2). Among 18 cultures, 10 culture extracts showed inhibitory zones although no growth of strain KA20 was observed. For example, the fed culture of *Streptomyces venezuelae* ATCC15439 caused the growth inhibition of KA20, and a considerable inhibitory zone (2.65 cm) against *M. luteus* (Table S2). This strain accumulates a significant amount of pikromycin [43], which led to the growth inhibition of both strain KA20 and *M. luteus*. Remarkably, 8 strains (HUT6003, HUT6072, HUT6098, HUT6168, HUT6196, JCM4980, NBRC12849, and GK7) showed inhibitory zone without growth inhibition of strain KA20 (Table S2). ESI-MS analysis of these 8 fed cultures revealed that all activities were not related to the restoration LC and LM in strain KA20 (Fig. S4). For example, the fed culture of strain HUT6168 showed a distinct ion peak at *m*/*z* 787.46 [Fig. S4-(9)], which was consistent with that of dinactin, a member of macrotetrolides. Its biosynthetic genes [44–46] could not be detected in an *S. rochei* genome [20], indicating the antimicrobial activity against *M. luteus* was not caused by the extract of KA20. As shown in Fig. S4-(14), a fed culture of *S. cellostaticus* NBRC12849 showed the presence of a considerable amount of LM. Strain NBRC12849 is known to be a producer of LC and LM, therefore, the presence of inducing molecules such as SRBs was investigated.

We tested whether *Streptomyces cellostaticus* NBRC12849, a LC and LM producer, have an ability to accumulate SRBs as antibiotic inducers. As described above, its fed sample showed inhibitory zone against *M. luteus*, however, strain NBRC12849 accumulate LC and LM as their original secondary metabolites. Hence, the culture extract at an early-growth-phase was partially separated from LC and LM by Sephadex LH20, and then the fractions were fed into strain KA20 (Fig. S5). As shown in Fig. S5a, antibiotic production in KA20 was restored by the feeding of the LH20 fractions of *S. cellostaticus* NBRC12849. Furthermore, the corresponding ESI-MS peaks for SRB1 and SRB2 were detected in fraction No.23, which fraction has an inducing activity for LC and LM (Fig. S5b). This finding indicated that *S. cellostaticus* NBRC12849 accumulate SRBs as antibiotic inducers. The amino-acid sequence homology of AQI88_RS40210 protein in *S. cellostaticus*. has 86% identity with SrrX in *S. rochei* 7434AN4.

**Fig. 3:**
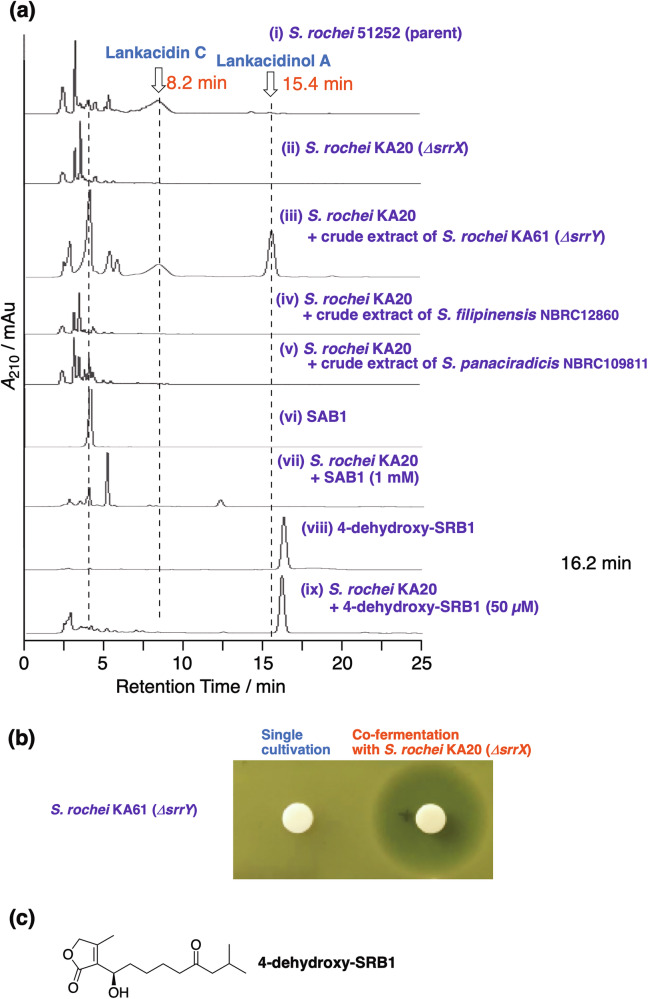
Structural diversity of SRB-type butenolides using an antibiotic production restoration assay in *S. rochei* strain KA20 (*srrX*-deficient mutant of *S. rochei* 51252). **a** Representative data in a feeding experiment, evaluated through HPLC analysis. (i) Strain 51252 (parent), (ii) strain KA20, (iii) strain KA20 supplemented the crude extract of strain KA61 (*srrY*-deficient mutant of *S. rochei* 51252), (iv) strain KA20 supplemented the crude extract of *S. filipinensis* NBRC 12860, (v) strain KA20 supplemented the crude extract of *S. panaciradicis* NBRC 109811, (vi) synthetic SAB1, (vii) strain KA20 supplemented with synthetic SAB1 (1 mM), (viii) 4-dehydroxy-SRB1, (ix) strain KA20 supplemented with 4-dehydroxy-SRB1 (50 µM). **b** Co-fermentation experiment, evaluated through bioassay using *Micrococcus luteus* as an indicator microorganism. Single cultivation of the recipient strains and co-fermentation of the recipients with strain KA20 were performed, and their inhibitory zones were analyzed. Positive control using strain KA61 was shown in this panel (other data was shown in Tables S1 and S3). **c** Structure of 4-dehydroxy-SRB1 accumulated by a feeding of synthetic SRB1 in strain KA20

Some representative HPLC spectra were shown in Fig. 3a. Restoration of lankacidin antibiotics (Fig. S2), lankacidin C (retention time; 8.2 min) and lankacidinol A (15.4 min), was monitored by HPLC at 230 nm [column; Nacalai Tesque Cholester (4.6 mm I.D. x 250 mm), eluent; acetonitrile–50 mM sodium phosphate buffer (pH 8.2) = 3:7 (v/v), and a flow rate; 1.0 mL/min]. Strain KA20 failed to produce LC and LM due to the deficiency of *srrX* [Fig. 3a-ii and Fig. S4-(20)], whereas the feeding of the KA61 extract into strain KA20 restored LC and LM production [Fig. 3a-iii and Fig. S4-(19)]. Remarkably, no other metabolites were detected in a feeding of SRB into strain KA20, which indicates that SRB is an inducer for two antibiotics, LC and LM, in *S. rochei*. We further investigated whether two strains, *S. filipinensis* NBRC12860 and *S. panacoiradicis* NBRC109811, both harbor the highly homologous gene products with SrrX (55% identity), could restore LC and LM production in KA20. These two extracts showed no ability to restore antibiotic production in KA20 (Fig. 3a-iv, -v), suggesting that these strains may have different type of signaling molecules or are free of a signaling-molecule-dependent regulation.

The feeding experiment has a disadvantage on detection of water-soluble SRB-type molecules (for example, phosphorylated biosynthetic intermediates) since the culture extracts were obtained through EtOAc extraction. In addition, insufficient amount of culture extracts from donor strains may fail the restoration of LC and LM production. Remarkably, growth inhibition of the recipient strain KA20 occurred even when the culture extract from donor strains with two-fold equivalent, possible due to the presence of growth-inhibitory factors such as antibiotics produced by donors.

To solve these problems, we focused on a co-fermentation strategy, which could lead to continuous transfer of signaling molecule(s) from the donor strains to the recipient strain KA20 during the cultivation. Co-fermentation of 122 donor strains with strain KA20 was performed, and the restoration of LC and LM production was evaluated by an increase of inhibitory zone in the co-fermentation samples when compared with their single cultivation. As anticipated, co-fermentation of *S. rochei* strain KA61 with strain KA20 (as a positive control) led to increase an inhibitory zone against *M. luteus* (Fig. 3b and Table S3). Increase of inhibitory zone in co-fermentation when compared with single cultivation was observed in 8 strains, including HUT6021, HUT6072, HUT6166, HUT6228, JCM5042, Tü113, NBRC13447, and TOHO-M025 (Tables S1 and S3). Their ESI-MS analysis was performed to judge whether the growth inhibition against *M. luteus* was due to the restoration of LC and LM through the cell-to-cell transfer of SRB-type molecules from the donors to strain KA20 (Table S3 and Fig. S6). The corresponding peaks for LC and LM were not detected among the 8 co-fermented samples described above, suggesting that increase of antimicrobial activity in their co-culture samples was not due to induction by SRB-type molecules. Co-fermentation of strain Tü113 with KA20 showed a distinct peak at *m*/*z* 795.34 [Fig. S6-(5)], which was not detected in a single cultivation and the fed culture. This finding reminds us the following possibilities; (1) a transfer of water-soluble inducing factor from Tü113 to KA20, (2) a transfer of inducing factor from KA20 to Tü113, or (3) some building block(s) accumulated in a donor strain would secrete to a recipient strain to construct the antimicrobial compound with *m*/*z* 795.34. At this moment, we could not identify the compound corresponding to the distinct peak at *m*/*z* 795.34, which will be elucidated in due course. Regarding third possibility, Kurosawa et al. reported an outstanding example of bacterial-bacterial communication; co-fermentation of *Rhodococcus fascians* and *Streptomyces padanus* led to synthesize a new type of antibiotics, rhodostreptomycins [47]. These antibiotics were biosynthesized in *Rhodococcus* harboring megaplasmid, which was generated through a horizontal transfer from *Streptomyces padanus*. Thus, competing bacteria through a transfer of inducing elements (signaling molecules), building blocks, and biosynthetic genes/proteins, may have benefits to acquire new items to survive under stress environment.

Combined with feeding and co-fermentation experiments among 122 donor strains, an inducing activity of LC and LM was detected only in *S. cellostaticus* NBRC12849, a producer of LC and LM. Thus, SRB-type molecules are rare distribution in *Streptomyces* species.

### Evaluation of antibiotic-inducing activity of SRB analog, SAB1, in *S. rochei*

**Fig. 4:**
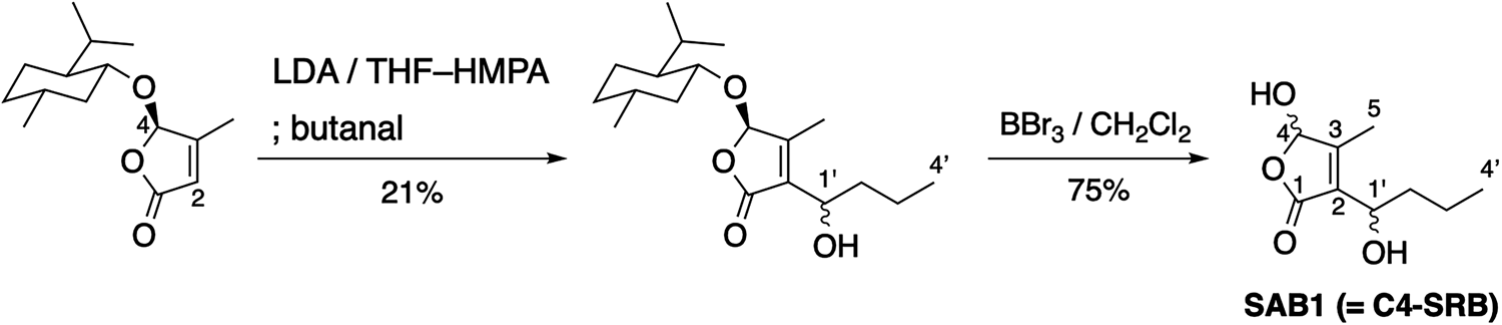
Synthetic scheme of an SRB analog, SAB1 ( = C4-SRB)

Wang and co-workers demonstrated the isolation of SRB-type butenolide molecules SAB1–3 that are responsible for inducers of nikkomycin production in *S. ansochromogenes* (Fig. S1) [18, 48]. Nevertheless its biosynthetic enzyme SabA produces SRB-type butenolides, its amino acid sequence has moderate similarity/identity to SrrX (42% identity and 77% similarity), and located on an different clade, Clade VIII, as shown in Fig. 2. To evaluate their inducing activity in *S. rochei*, we synthesized SAB1 (Fig. 1 and S1) in a similar manipulation for the synthesis of SRBs (Fig. 4) [16, 17]. The commercially available *n*-butanal was coupled with enantiomerically pure 3-methyl-4-(L-menthyloxy)but-2-en-1,4-olide [16, 17, 40, 41] in the presence of lithium diisopropylamide (LDA) to give a diastereomeric mixture of butenolides in the ratio 1:1 in 21% yield. Since the C-1’ configuration of SAB1 has not yet been determined [18], we attempted deprotection by BBr_3_ without separation of these diastereomers to give racemic SAB1 ( = C4-SRB) in 75% yield.

Synthetic SAB1 (a C_4_ side chain at C-2 of butenolide) showed no inducing activity even at 1 mM concentration in strain KA20 (Fig. 3a-vi and -vii). In our preceding results, 6’-deoxo-SRBs (C_12_ or C_13_ side chain at C-2 of butenolide) (Fig. S3), products of *srrO*-deficient strain KA54, showed ligand-binding activity (Teshima et al. 2020). These findings indicate the importance of the chain length at C-2 of butenolides.

### Detection and evaluation of antibiotic-inducing activity of SRB analog, 4-dehydroxy-SRB1, in *S. rochei*

In a feeding of SRB1 into strain KA20, we noticed the accumulation of a greenish spot at Rf = 0.7 in CHCl_3_–MeOH (10:1, v/v) on TLC after anisaldehyde staining and baking. This compound was purified and structure elucidated. ESI-MS analysis demonstrated to be C_15_H_24_O_4_, whose molecular formula was one oxygen atom smaller than that of SRB1. In its NMR spectra, hemiacetal methine at C-4 in SRB1 was changed to methylene, and other signals were well conserved in those of SRB1. Thus, this compound was determined to be 4-dehydroxy-SRB1 as a novel compound (Fig. 3c). We then fed 4-dehydroxy-SRB1 into strain KA20, however, the fed culture failed to restore LC and LM production even at 1000-fold higher concentration (50 µM), compared with a minimum antibiotic-inducing concentration of SRB1 (40 nM) (Fig. 3a-viii and -ix), suggesting the functional importance of a C-4 hydroxyl group for inducing activity in *S. rochei*. Remarkably, detection of 4-dehydroxy-SRB1 in the culture extract of *S. rochei* reminds us to propose signaling-molecule quenching system in *Streptomyces*.

**Fig. 5:**
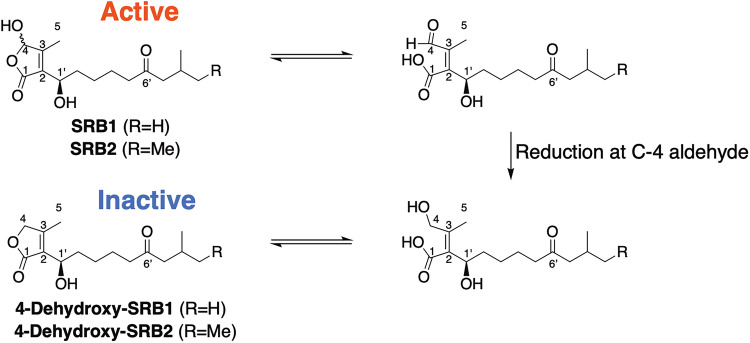
Possible signaling-molecule quenching mechanism of the formation of “inactive” SRB derivatives, 4-dehydroxy-SRBs, from “active” SRBs in *Streptomyces rochei* 7434AN4

Biogenesis of 4-dehydroxy-SRB1 is proposed as follows (Fig. 5); a hemiacetal ring at C-4 in SRB1 was opened, and then subsequent reduction and ring-closure occurred to form 4-dehydroxy-SRB1, which was first discovered in nature. We have previously isolated “active” signaling molecules SRBs with a help of bioassay in *S. rochei* KA20 (average yield; 6 µg L^–1^) [16], hence, this is a first discovery of an “inactive” signaling molecule in the culture. Detection of “inactive” 4-dehydroxy-SRBs may be related to a signaling-molecule “quenching” system in *Streptomyces* species. In gram negative bacteria, *N*-acyl-homoserine lactones (AHLs) act as “quorum sensing” autoinducers to activate gene expression for bioactive materials including virulence toxins and biofilm [49]. Inhibition of quorum sensing, so-called “quorum quenching”, is nowadays well investigated in pharmaceutical application. Quorum sensing inhibition does not affect the cell growth, which in turn minimizes the occurrence drug-resistant bacteria [50]. In *Streptomyces*, quenching of signaling molecules may lead to abolish antibiotic production in the original strain. This scenario is plausible for the use of nutrients on energy metabolism, cell division, signaling-molecule-independent metabolite production, and protease secretion for bioremediation. Conversion of “active” SRBs into “inactive” 4-dehydroxy-SRBs occurred in the culture broth of *S. rochei*, indicating the presence of enzyme(s) for signaling-molecule “quenching” system, which will be further investigated in due course in our laboratory.

## Conclusion

*Streptomyces* signaling molecules act as inducers for secondary metabolite biosynthesis by binding to their cognate receptors to dissociate the signaling-molecules/receptors complex from their target nucleotide sequences [51, 52]. Our present finding revealed a rare distribution of SRB-type butenolides among the *Streptomyces* signaling molecules. Many *Streptomyces* strains contain more than 30 secondary metabolites biosynthetic gene clusters on their genome, however, many of them (around 80-90%) are silent or at low transcription level under normal laboratory culture conditions. Manipulation of regulatory genes often causes activation of “silent” secondary metabolites [5, 53–55]. Among 40 biosynthetic gene clusters (35 in the chromosome and 5 in pSLA2-L) in *S. rochei* 7434AN4, only six compounds are structurally confirmed in a normal laboratory culture condition [20]. Our group has identified various secondary metabolites by genome mining through genetic manipulation [56–59]. Remarkably, *Streptomyces* strains generally have multiple signaling-molecule receptor genes, for example, *S. rochei* 7434AN4 has two signaling-molecule biosynthetic genes (*srrX* on a linear plasmid pSLA2-L and *SRO_3382* on the chromosome) and seven receptor homolog genes (*srrA–C* on pSLA2-L and 4 genes on the chromosome). Our group investigates the targets and ligands for receptors in *S. rochei*. SrrA protein binds to a promoter region of an SARP-type activator gene *srrY*, and its complex was dissociated by ligand molecules, SRBs, for LC and LM production [35]. SrrB protein is classified as a pseudo-receptor, and binds to the promoter of *srrY*, however, its gross ligands are uncertain (SRBs can bind but their sensitivity is less than thousand-folds compared with SrrA) [39]. A mutant of *srrC* produces LC and LM comparable to the parent strain, however, exhibits bald phenotype, indicating that SrrC protein has no direct function on antibiotic production. At this moment, we have not yet clarified the targets and ligands for SrrC, and other 4 receptor homologs coded on the *S. rochei* chromosome. Functional analysis of other receptors and another signaling-molecule biosynthesis protein SRO_3382 provides a great potential to recognize heterologous signaling molecules for activation of silent secondary metabolite biosynthetic gene clusters. Thus, SRB-type signaling molecules may act as heterologous inducers for secondary metabolite production in various *Streptomyces* strains through bacteria-bacteria communication, which will provide a versatile “genetic engineering-free” genome mining tool.

## Supplementary information

Supplemental Material_distribution of SRB.pdf
