# Supplementary material for: Rare distribution of butenolide-type signaling molecules among Streptomyces strains and functional importance as inducing factors for secondary metabolite production in Streptomyces rochei 7434AN4
Source: J Antibiot (Tokyo). 2025 Jun 12;78(8):488–99. doi: 10.1038/s41429-025-00840-9 (PMC12301235; doi:10.1038/s41429-025-00840-9)
Supplement: Supplementary file 1 — Supplemental Material_distribution of SRB.pdf [file 41429_2025_840_MOESM1_ESM.pdf]

SUPPLEMENTAL MATERIAL

Rare Distribution of Butenolide-type Signaling Molecules among *Streptomyces* Strains and Functional Importance as Inducing Factors for Secondary Metabolite Production in *Streptomyces rochei* 7434AN4

Asahi Hirata[1,2,†], Miho Sumiyoshi[3,†], Hazuki Fujita[2,4], Momoko Akimoto[1,2], Mary Hannah Rose A. Padayao[1,2], Yuto Eguchi[1], Maki Matsuura[5], Miyuki Otsuka[5], Kuninobu Inada[6], Aiko Teshima[1,2], and Kenji Arakawa[1-4,*]

[1] Program of Biotechnology, Graduate School of Integrated Sciences for Life, Hiroshima University, 1-3-1 Kagamiyama, Higashi-Hiroshima, Hiroshima 739-8530, Japan

[2] Hiroshima Research Center for Healthy Aging (HiHA), Hiroshima University, 1-3-1 Kagamiyama, Higashi-Hiroshima, Hiroshima 739-8530, Japan

[3] Department of Molecular Biotechnology, Graduate School of Advanced Sciences of Matter, Hiroshima University, 1-3-1 Kagamiyama, Higashi-Hiroshima, Hiroshima 739-8530, Japan

[4] Faculty of Engineering, Hiroshima University, 1-4-1 Kagamiyama, Higashi-Hiroshima, Hiroshima 739-8527, Japan

[5] College of Agriculture, Tamagawa University, 6-1-1 Tagawagagakuen, Machida, Tokyo 194-8610, Japan

[6] Natural Science Center for Basic Research and Development, Hiroshima University, 1-4-2 Kagamiyama, Higashi-Hiroshima, Hiroshima 739-8526, Japan

* Address correspondence to Kenji Arakawa, karakawa@hiroshima-u.ac.jp.

[†] These authors have contributed equally to this work.

Experimental Section

Isolation of *Streptomyces* species from soil

Fifteen strains (TU22 strain series) described in Table S1 were isolated as following procedure. Soil was collected from three spots; Area T01 (Okagami, Asao-ku, Kawasaki City, Tokyo, Japan), area K01 (Kushi, Bōnotsu Cho, Minami-Satsuma City, Kagoshima, Japan), and area H01 (Biruwa, Teshikaga Cho, Kawakami District, Hokkaido). Soil was obtained from 5-10 cm in depth in each area. Dried soil samples (1 g) were suspended in sterile water, and treated with 10-fold volume of 50 mM sodium phosphate buffer (pH 7.0) containing 0.05% sodium dodecyl sulfate (SDS) and 0.06% yeast extracts at 40°C for 20 min. This suspension was further diluted with 10-fold volume of 50 mM sodium phosphate buffer (pH 7.0), and then treated with 50 mg/mL lysozyme. Aliquot of the treated suspension (0.5 ml) was spread on Humic acid-vitamin agar (HV agar) [Ref.S1], and incubated at 30°C for 2 weeks. The generated colonies were further inoculated on ISP5 agar medium [Ref.S2], and incubated at 30°C for 2 weeks.

References:

S1) Hayakawa M, Nonomura H. 1987. Humic acid-vitamin agar, a new medium for the selective isolation of soil actinomycetes. *J Ferment Technol* 65:501-509 (1987).

S2) Shirling EB, Gottlieb D. 1966. Methods for characterization of *Streptomyces* species. *Int J Systemic Bacteriol* 16:313-340.

Table S1. *Streptomyces* strains used in this study

Strain ID	Other ID	Synonymous name	Inhibitory zone *1,2			Reference
			Feeding into KA20*3-5	Single culture*5	Mixed culture*5	
HUT 6001	NBRC3174	*Streptomyces antibioticus*	–	–	–	
HUT 6003	NBRC3303	*Streptomyces aureus*	+ (1.15)	+	+	
HUT 6021		*Streptomyces* sp.	–	–	+	
HUT 6022	NBRC3150	*Streptomyces olivaceus*	–	–	–	
HUT 6023	NBRC3152	*Streptomyces olivaceus*	–	–	–	
HUT 6024	NBRC3365	*Streptomyces venezuelae*	GI (–)	–	–	
HUT 6030	NBRC3113	*Streptomyces viridochromogenes*	–	–	–	
HUT 6031		*Streptomyces viridochromogenes*	–	+	–	
HUT 6034	NBRC3112	*Streptomyces verne*	–	–	–	
HUT 6035	NBRC3117	*Streptomyces antibioticus*	GI (1.98)	+	–	
HUT 6037		*Streptomyces griseus*	–	–	–	
HUT 6043		*Streptomyces sindenensis*	–	+	–	
HUT 6046	ATCC3381	*Streptomyces albus*	–	+	–	
HUT 6047	ATCC0618	*Streptomyces albus*	–	–	–	
HUT 6057	ATCC3325	*Streptomyces griseolus*	–	–	–	
HUT 6063		*Streptomyces purpurascens*	GI (1.17)	–	–	
HUT 6069	NBRC12819	*Streptomyces rutgersensis*	–	–	–	
HUT 6072	NIHJ213	*Streptomyces venezuelae*	+ (1.30)	–	+	
HUT 6076	NBRC3113	*Streptomyces viridochromogenes*	–	–	–	
HUT 6077		*Streptomyces cacaoi*	–	+	+	
HUT 6089		*Streptomyces pseudogriseolus*	–	–	–	
HUT 6090		*Streptomyces echinatus*	–	+	+	
HUT 6098	NBRC12768	*Streptomyces flaveolus*	+ (0.96)	–	–	
HUT 6100	NBRC12907	*Streptomyces rimosus*	GI (1.68)	+	+	
HUT 6105		*Streptomyces griseus*	–	–	–	
HUT 6106		*Streptomyces griseus*	–	–	–	
HUT 6121		*Streptomyces griseus*	–	–	–	
HUT 6124		*Streptomyces bostroemi*	–	–	–	
HUT 6126		*Streptomyces albidoflavus*	–	–	–	
HUT 6127		*Streptomyces albidoflavus*	–	–	–	
HUT 6128	IPV875	*Streptomyces albidoflavus*	–	–	–	
HUT 6131		*Streptomyces albus*	GI (1.28)	–	–	
HUT 6134		*Streptomyces albus*	–	–	–	

HUT 6135		*Streptomyces albus*	–	–	–	
HUT 6138	IPV551X	*Streptomyces aureofaciens*	–	–	–	
HUT 6143		*Streptomyces citreus*	–	–	–	
HUT 6148	IPV106X	*Streptomyces flavus*	–	+	–	
HUT 6150		*Streptomyces fulvoviridis*	–	–	–	
HUT 6154		*Streptomyces griseoroseus*	GI (1.25)	+	–	
HUT 6155		*Streptomyces halstedii*	–	–	–	
HUT 6158		*Streptomyces novaecaesareae*	–	–	–	
HUT 6162	IPV611	*Streptomyces roseus*	–	+	–	
HUT 6163		*Streptomyces roseus*	–	–	–	
HUT 6166		*Streptomyces viridochromogenes*	–	–	+	
HUT 6167	IPV973	*Streptomyces viridochromogenes*	–	–	–	
HUT 6168	IPV646	*Streptomyces vulgaris*	+ (1.57)	+	–	
HUT 6172	OUT8322	*Streptomyces fradiae*	–	–	–	
HUT 6174	OUT8325	*Streptomyces fradiae*	–	–	–	
HUT 6175	OUT8326	*Streptomyces fradiae*	–	–	–	
HUT 6177	OUT8328	*Streptomyces fradiae*	–	–	–	
HUT 6178		*Streptomyces griseus*	–	–	–	
HUT 6186		*Streptomyces griseus*	–	–	–	
HUT 6190		*Streptomyces lusitanus*	–	+	–	
HUT 6194		*Streptomyces humidus*	–	–	–	
HUT 6196		*Streptomyces agglomeratus*	+ (2.20)	–	–	
HUT 6197		*Streptomyces ahygroscopicus*	–	+	–	
HUT 6199		*Streptomyces atrocyaneus*	–	–	–	
HUT 6201		*Streptomyces aurantiacogriseus*	–	–	–	
HUT 6202		*Streptomyces castaneoglobisporus*	–	–	–	
HUT 6204		*Streptomyces flavomacrosporus*	–	–	–	
HUT 6207		*Streptomyces hygrospinosus*	GI (–)	+	–	
HUT 6216		*Streptomyces rubrolavendulae*	–	+	–	
HUT 6225		*Streptomyces hiroshimensis*	–	–	–	
HUT 6227		*Streptomyces purpurogeniscleroticus*	–	–	–	
HUT 6228		*Streptomyces sclerotialus*	–	–	+	
HUT 6259	NBRC3430	*Streptomyces griseus*	–	–	–	
HUT 6263		*Streptomyces lavendulae*	–	–	–	
HUT 6264		*Streptomyces flavotricini*	–	–	–	

HUT 6269	NBRC12851	*Streptomyces chromofuscus*	–	–	–	
HUT 6276		*Streptomyces lividans*	–	–	–	
HUT 6613		*Streptomyces albus*	–	–	–	
HUT 6624		*Streptomyces albus*	GI (–)	+	–	
JCM 4193		*Streptomyces ramulosus*	GI (–)	–	–	
JCM 4229		*Streptomyces griseus*	–	–	–	
JCM 4276		*Streptomyces griseofuscus*		+	+	
JCM 4623		*Streptomyces griseus*	–	–	–	
JCM 4979		*Streptomyces violaceoruber*	GI (–)	+	–	
JCM 4980		*Streptomyces antibioticus*	+ (2.37)	+	–	
JCM 5042		*Streptomyces panayensis*	–	–	+	
JCM 5070		*Streptomyces avermitilis*	–	+	–	
JCM 7250		*Streptomyces papulosa*	–	–	–	
TU 113		*Streptomyces parvulus*	GI (1.38)	–	+	
TU 4055		*Streptomyces parvulus*	GI (2.74)	+	–	
NBRC 12517		*Streptomyces yerevanensis*	GI (–)	+	+	
NBRC 12779		*Streptomyces griseoplanus*	–	–	–	
NBRC 12800		*Streptomyces mutabilis*	–	–	–	
NBRC 12809		*Streptomyces prasinopilosus*	–	–	–	
NBRC 12836		*Streptomyces ambofaciens*	GI (–)	–	–	
NBRC 12849		*Streptomyces cellostaticus*	+ (3.00)	+	+	
NBRC 12860		*Streptomyces filipinensis*	–	–	–	
NBRC 12866		*Streptomyces gelaticus*	–	–	–	
NBRC 13108		*Streptomyces yokosukanensis*	–	+	–	
NBRC 13352		*Streptomyces viridifaciens*	–	–	–	
NBRC 13407		*Streptomyces rishiriensis*	–	–	–	
NBRC 13447		*Streptomyces katrae*	–	–	+	
NBRC 13448		*Streptomyces regensis*	+ (2.28)	+	–	
NBRC 13472		*Streptomyces hygroscopicus* subsp. *hygroscopicus*	–	+	–	
IFO 14057		*Streptomyces cattleya*	GI (–)	–	–	
IFO 14059	NBRC14059	*Streptomyces griseus*	–			
NBRC109811		*Streptomyces panaciradicis*	–	–	–	
M145		*Streptomyces coelicolor*	–	–	–	
ATCC15439		*Streptomyces venezuelae*	GI (2.65)	+	+	
GK 3		*Streptomyces* sp.	–	+	–	Cao et al. 2012

GK 7		*Streptomyces* sp.	+ (1.43)	+	–	Ref. S3
GK 15		*Streptomyces* sp.	–	–	–	Ref. S3
GK 18		*Streptomyces* sp.	GI (1.12)	+	–	Ref. S3
TU22T01N01		*Streptomyces* sp.	–	+	–	This study
TU22T01N02		*Streptomyces* sp.	–	–	–	This study
TU22T01N06		*Streptomyces* sp.	–	–	–	This study
TU22T01N08		*Streptomyces* sp.	–	+	–	This study
TU22T01N10		*Streptomyces* sp.	–	–	–	This study
TU22T01N13		*Streptomyces* sp.	–	–	–	This study
TU22T01N17		*Streptomyces* sp.	–	–	–	This study
TU22K01L01		*Streptomyces* sp.	–	–	–	This study
TU22K01L02		*Streptomyces* sp.	–	–	–	This study
TU22K02L03		*Streptomyces* sp.	–	–	–	This study
TU22K02L09		*Streptomyces* sp.	–	+	–	This study
TU22H01L01		*Streptomyces* sp.	–	+	–	This study
TU22H01L02		*Streptomyces* sp.	–	–	–	This study
TU22H03L01		*Streptomyces* sp.	–	–	–	This study
TU22H03L02		*Streptomyces* sp.	–	–	–	This study
TOHO-M025		*Streptomyces* sp.	–	–	+	Ref. S4
51252		*Streptomyces rochei* derivative with pSLA2-L	+ (1.20)	+	+	Ref. S5
KA20		*Streptomyces rochei* mutant with *srrX* deletion	–	–	–	Ref. S6
KA61		*Streptomyces rochei* mutant with *srrY* deletion	+ (2.59)	–	+	Ref. S7

*1 Diameter of paper disk was 0.8 cm.

*2 *Micrococcus luteus* was used as an indicator microorganism.

*3 GI indicates growth inhibition of strain KA20 in the presence of the culture extracts.

*4 The value in parenthesis indicates a size of inhibitory zone

*5 "+" indicates positive inhibitory zone, while "–" indicates negative inhibitory zone.

References

(S3) Cao Z, Khodakaramian G, Arakawa K, Kinashi H. Isolation of borrelidin as a phytotoxic compound from a potato pathogenic *Streptomyces* strain. *Biosci. Biotechnol. Biochem.*2012;76:353-357.

(S4) Fukumoto A, Murakami C, Anzai Y, Kato F. Maniwamycins: new quorum-sensing inhibitors against *Chromobacterium violaceum* CV026 were isolated from *Streptomyces* sp. TOHO-M025 *J Antibiot.* 1994;47:1447–1455.

(S5) Kinashi H, Mori E, Hatani A, Nimi O. Isolation and characterization of large linear plasmids from lankacidin-producing *Streptomyces* species. *J Antibiot.* 1994;47:1447–1455.

(S6) Arakawa K, Mochizuki S, Yamada K, Noma T, Kinashi H. Gamma-Butyrolactone autoregulator-receptor system involved in lankacidin and lankamycin production and morphological differentiation in *Streptomyces rochei*. *Microbiol.* 2007;153:1817–1827.

(S7) Yamamoto S, He Y, Arakawa K, Kinashi H. Gamma-Butyrolactone-dependent expression of the SARP gene *srrY* plays a central role in the regulatory cascade leading to lankacidin and lankamycin production in *Streptomyces rochei*. *J Bacteriol.* 2008;190:1308–1316.

Table S2. Strains with inhibitory zone when their culture extracts were fed into *S. rochei* KA20.

Strain entry	Strain ID	Strain name	Inhibitory zones [cm] *1,2	Lankacidins or Lankamycin on ESI-MS *3
1	HUT6003	*Streptomyces aureus*	1.15	N.D.
2	HUT6035	*Streptomyces antibioticus*	Growth inhibition / 1.98	N.D.
3	HUT6063	*Streptomyces purpurascens*	Growth inhibition / 1.17	N.D.
4	HUT6072	*Streptomyces venezuelae*	1.30	N.D.
5	HUT6098	*Streptomyces flaveolus*	0.96	N.D.
6	HUT6100	*Streptomyces rimosus*	Growth inhibition / 1.68	N.D.
7	HUT6131	*Streptomyces albus*	Growth inhibition / 1.28	N.D.
8	HUT6154	*Streptomyces griseoroseus*	Growth inhibition / 1.25	N.D.
9	HUT6168	*Streptomyces vulgaris*	1.57	N.D.
10	HUT6196	*Streptomyces agglomeratus*	2.20	N.D.
11	JCM4980	*Streptomyces antibioticus*	2.37	N.D.
12	Tü113	*Streptomyces parvulus*	Growth inhibition / 1.38	N.D.
13	Tü4055	*Streptomyces parvulus*	Growth inhibition / 2.74	N.D.
14	NBRC12849	*Streptomyces cellostaticus*	3.00	lankamycin
15	NBRC13448	*Streptomyces regensis*	2.28	N.D.
16	ATCC15439	*Streptomyces venezuelae*	Growth inhibition / 2.65	N.D.
17	GK7	*Streptomyces* sp.	1.43	N.D.
18	GK18	*Streptomyces* sp.	Growth inhibition / 1.12	N.D.
19	KA61 (Positive control)	*Streptomyces rochei* KA61	2.59	lankamycin, lankacidinol, lankacidinol A
20	KA20 (Negative control)			

*1 Diameter of paper disk was 0.8 cm.

*2 *Micrococcus luteus* was used as an indicator microorganism.

*3 Corresponding m/z values for lankacidins and lankamycin are follows;

lankamycin $[M+Na]^+$ = 855.47, lankacidinol A $[M+Na]^+$ = 526.24, lankacidin C $[M+Na]^+$ = 482.23, lankacidinol $[M+Na]^+$ = 484.23

Table S3. Strains with higher antimicrobial activity when co-fermentated with *S. rochei* KA20.

Strain entry	Strain ID	Strain name	Inhibitory zones [cm] *1,2	Lankacidins or Lankamycin on ESI-MS *4
1	HUT6021	*Streptomyces griseus*	(+) 1.0 (–) ND *3	N.D.
2	HUT6072	*Streptomyces venezuelae*	(+) 1.3 (–) 1.2	N.D.
3	HUT6166	*Streptomyces viridochromogenes*	(+) 2.4 (–) 1.6	N.D.
4	HUT6228	*Streptomyces sclerotialus*	(+) 1.0 (–) ND	N.D.
5	Tü113	*Streptomyces parvulus*	(+) 2.4 (–) 1.2	N.D.
6	JCM5042	*Streptomyces panayensis*	(+) 3.0 (–) 2.4	N.D.
7	NBRC13447	*Streptomyces katrae*	(+) 2.0 (–) ND	N.D.
8	TOHO-M025	*Streptomyces* sp. TOHO-M025	(+) 1.2 (–) ND	N.D.
9	KA61 (Positive control)	*Streptomyces rochei* KA61	(+) 2.3 (–) ND	lankamycin, lankacidinol A

*1 Hole size for samples was 0.8 cm.
*2 *Micrococcus luteus* was used as an indicator microorganism.
*3 (+) indicates the donor strain co-fermentated with strain KA20, while (–) indicates single cultivation of the donor strain.
*4 Corresponding m/z values for lankacidins and lankamycin are follows;
lankamycin $[M+Na]^+$ = 855.47, lankacidinol A $[M+Na]^+$ = 526.24, lankacidin C $[M+Na]^+$ = 482.23, lankacidinol $[M+Na]^+$ = 484.23

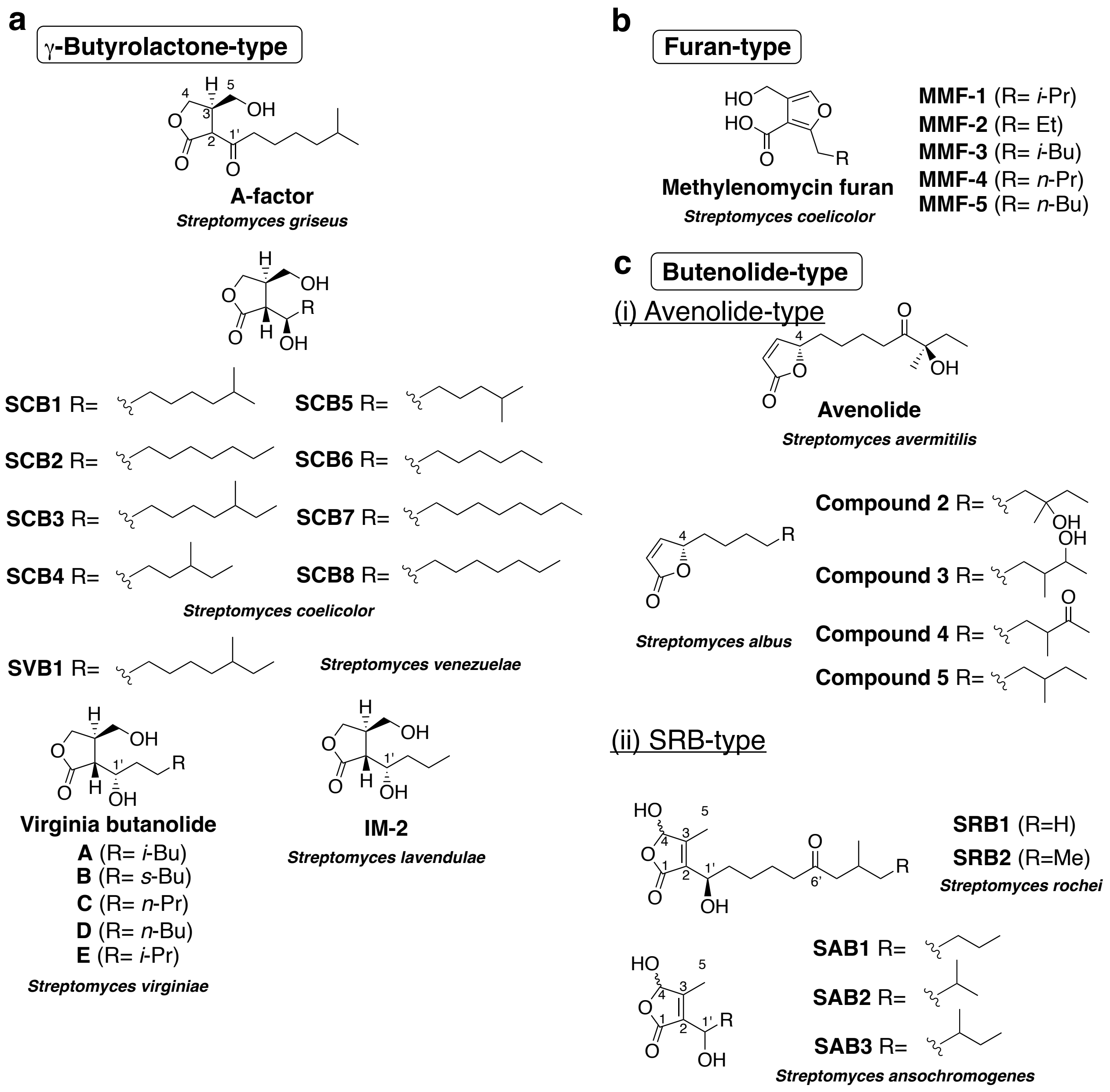

Figure S1. *Streptomyces* signaling molecules. **a**) γ-Butyrolactone-type molecules. A-factor from *Streptomyces griseus*, SCBs from *Streptomyces coelicolor*, virginia butanolides from *Streptomyces virginiae*, and IM-2 from *Streptomyces lavendulae.* **b**) Furan-type molecules. Methylenomycin furans from *Streptomyces coelicolor.* **c**) Butenolide-type moecules. (i) Avenolide-type butenolides. Avenolide from *Streptomyces avermectinius.* Compounds 2–5 from *Streptomyces albus*. (ii) SRB-type butenolides. SRB1 and SRB2 from *S. rochei* 7434AN4, and SRB analogs, SABs, from *Streptomyces ansochromogenes*. *i*-Bu, *iso*-butyl; *s*-Bu, *sec*-butyl; *n*-Pr; *normal*-propyl; *n*-Bu, *normal*-butyl; *i*-Pr, *iso*-propyl; Et, ethyl; Me, methyl.

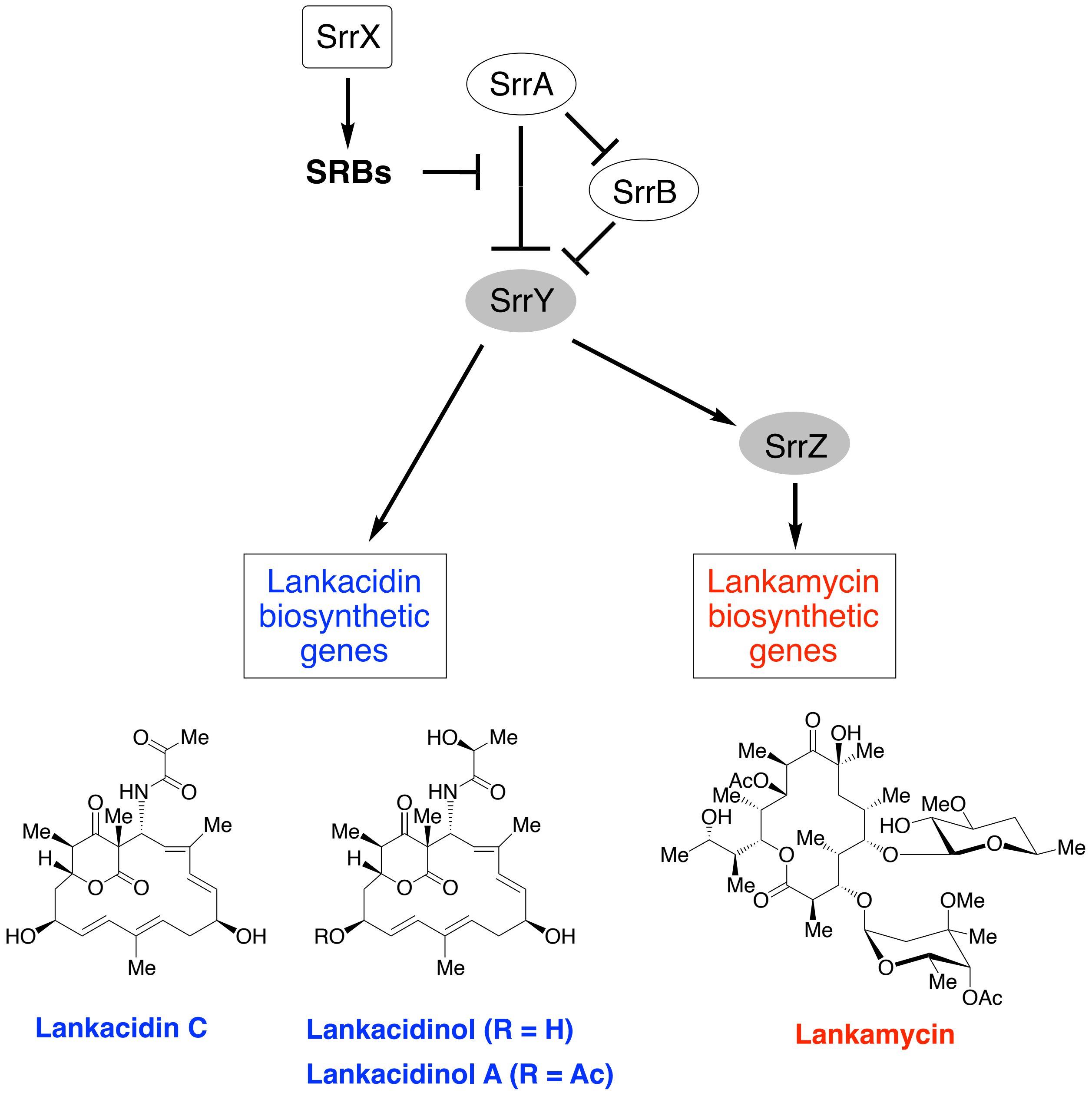

Figure S2. Regulatory cascade for lankacidin C, lankacidinol A, and lankamycin production in *Streptomyces rochei* 7434AN4. SrrX, SRB biosynthetic enzyme; SrrA, SRB receptor; SrrY and SrrZ, SARP-type activators; SrrB, pseudo-receptor that negatively regulates antibiotic production.

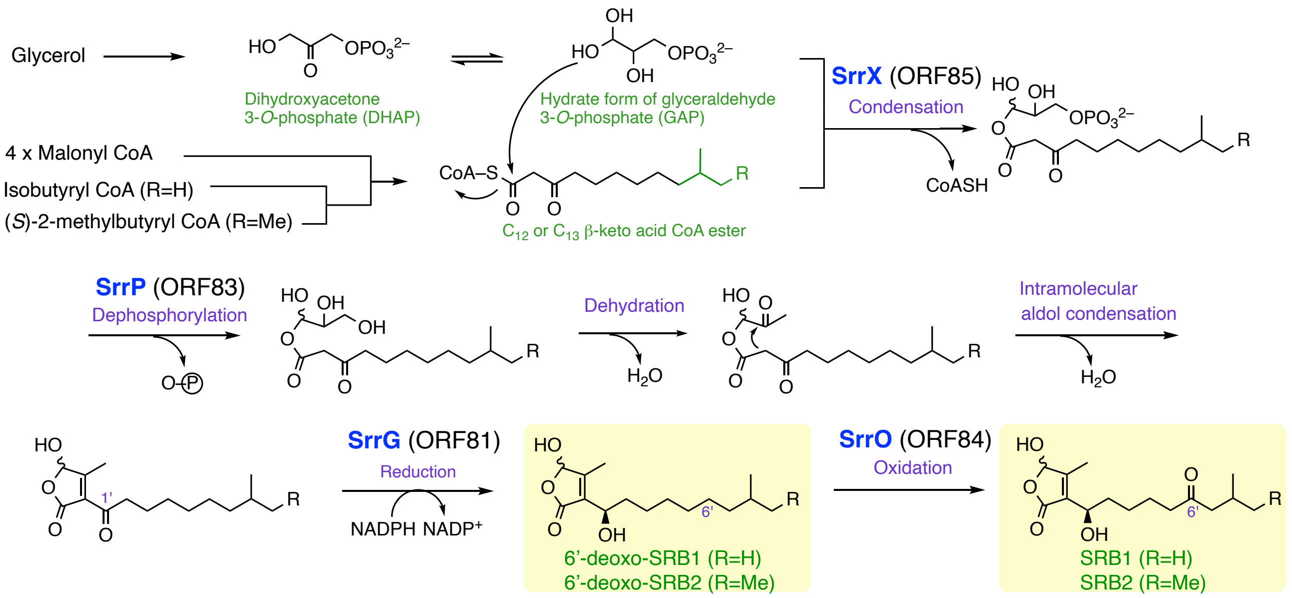

Figure S3. The possible biosynthetic pathway of SRBs in *S. rochei*. Me; methyl.

Figure S4. ESI-MS spectra of the fed culture of *S. rochei* KA20. The followings are 18 culture strains exhibiting inhibitory zones when their culture extracts were fed into *S. rochei* KA20 [(1) HUT6003, (2) HUT6035, (3) HUT6063, (4) HUT6072, (5) HUT6098, (6) HUT6100, (7) HUT6131, (8) HUT6154, (9) HUT6168, (10) HUT6196, (11) JCM4980, (12) Tü113, (13) Tü4055, (14) NBRC12849, (15), NBRC 13448, (16) ATCC15439, (17) GK7, and (18) GK18]. Positive control [panel (19)] and negative control [panel (20)] were also displayed. Detailed sample information was described in **Table S2**.

(1) HUT6003 extract in strain KA20

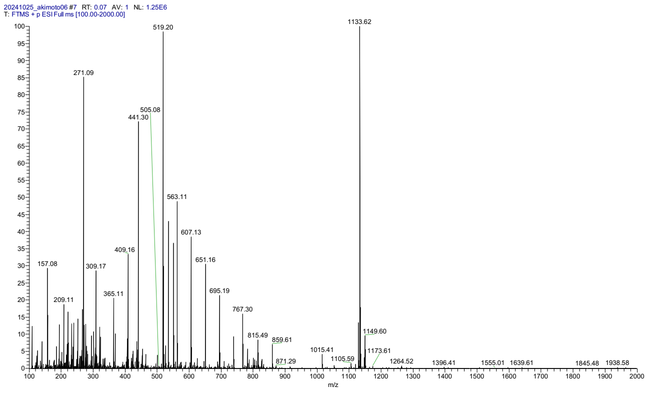

(2) HUT6035 extract in strain KA20

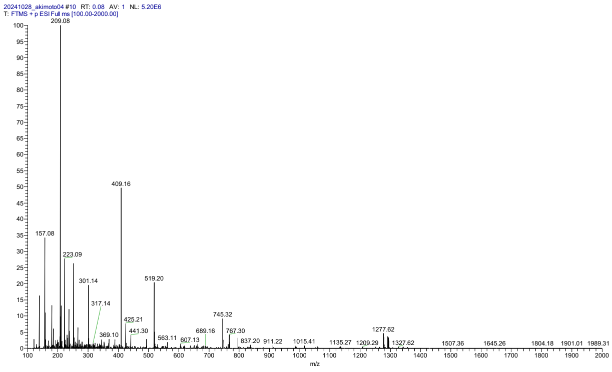

(3) HUT6063 extract in strain KA20

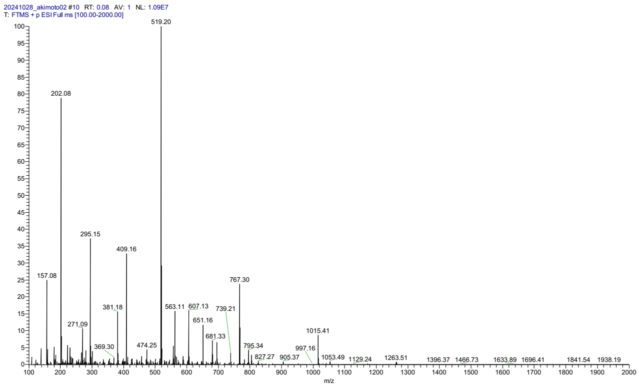

(4) HUT6072 extract in strain KA20

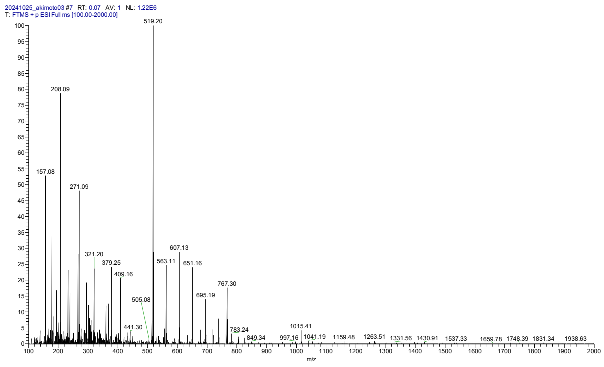

Figure S4. Continued.

(5) HUT6098 extract in strain KA20

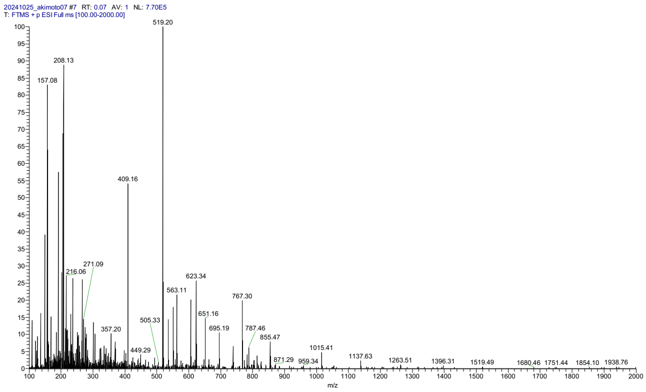

(6) HUT6100 extract in strain KA20

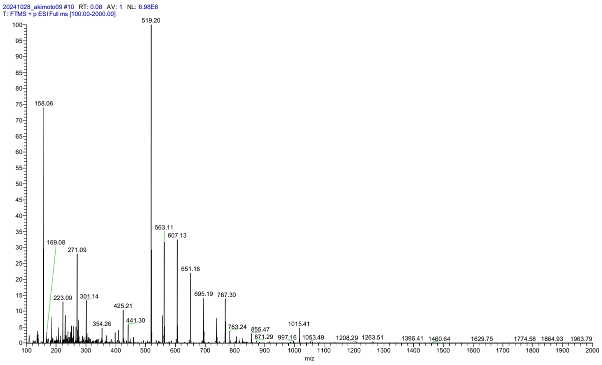

(7) HUT6131 extract in strain KA20

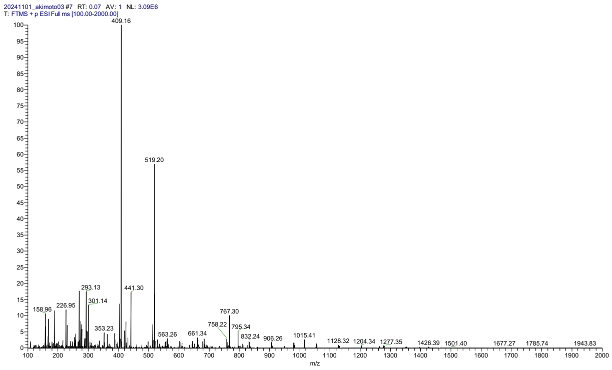

(8) HUT6154 extract in strain KA20

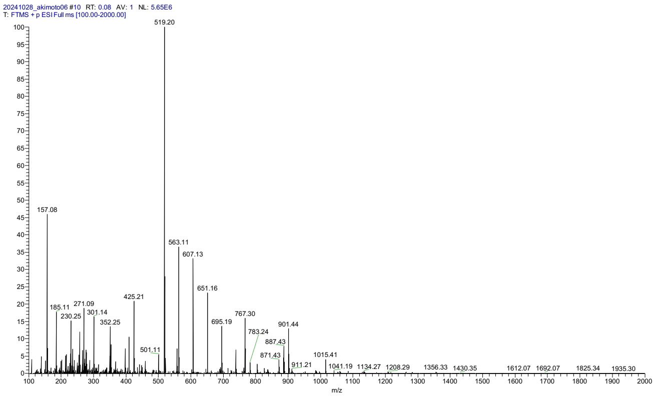

(9) HUT6168 extract in strain KA20

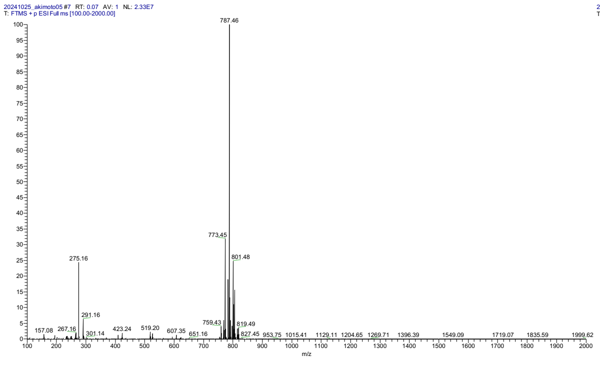

(10) HUT6196 extract in strain KA20

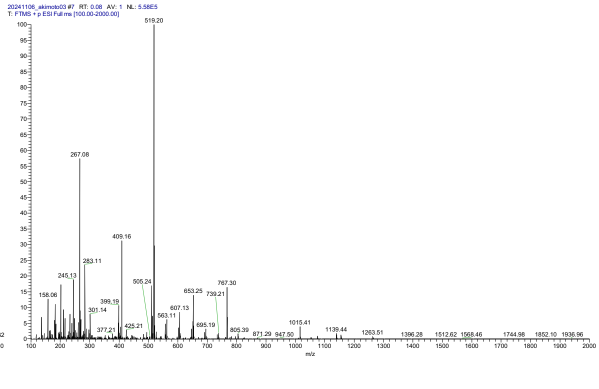

Figure S4. Continued.

(11) JCM4980 extract in strain KA20

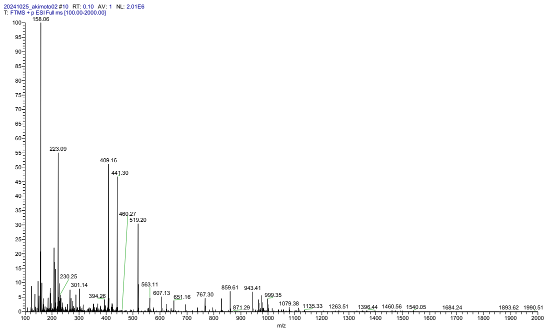

(12) Tü113 extract in strain KA20

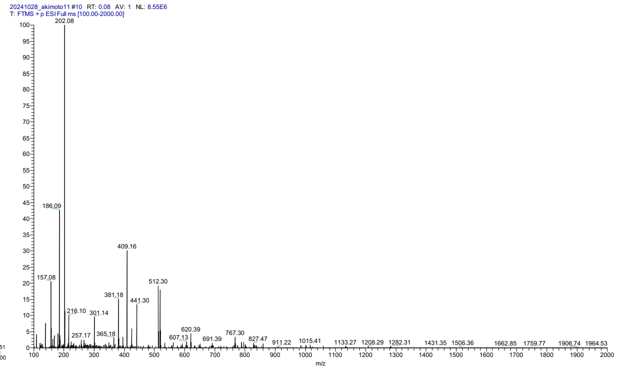

(13) Tü4055 extract in strain KA20

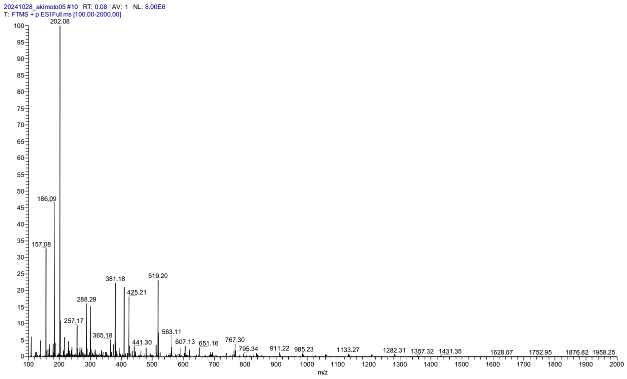

(14) NBRC12849 extract in strain KA20

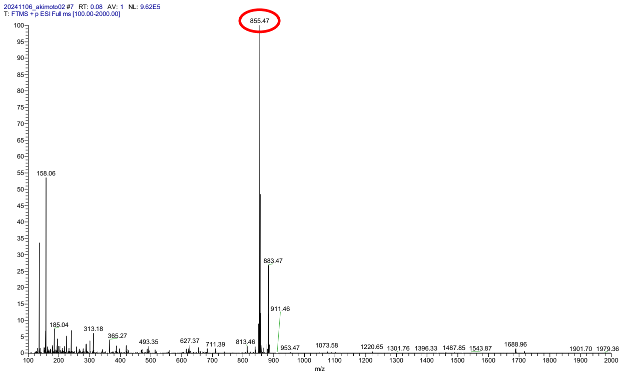

lankamycin [M+Na]$^+$ = 855.47

(15) NBRC13448 extract in strain KA20

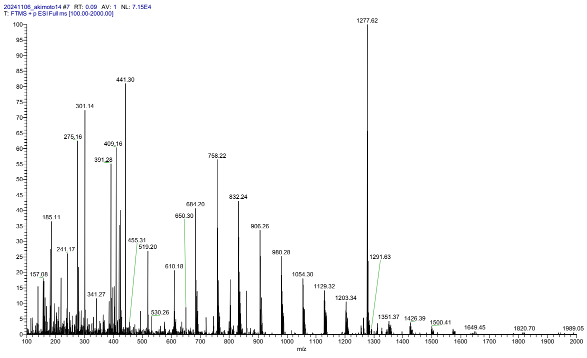

(16) ATCC15439 extract in strain KA20

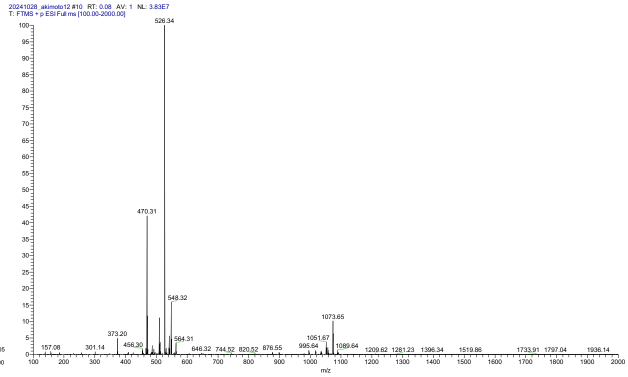

Figure S4. Continued.

(17) GK7 extract in strain KA20

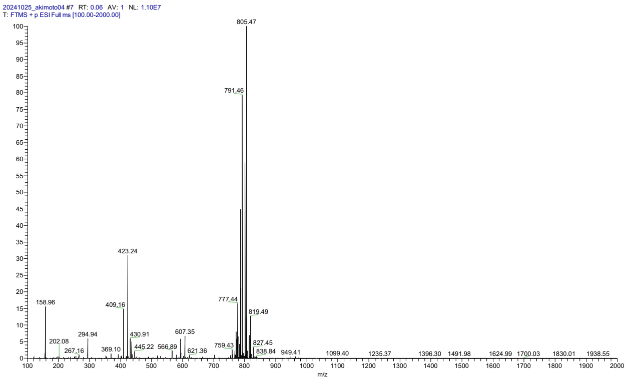

(18) GK18 extract in strain KA20

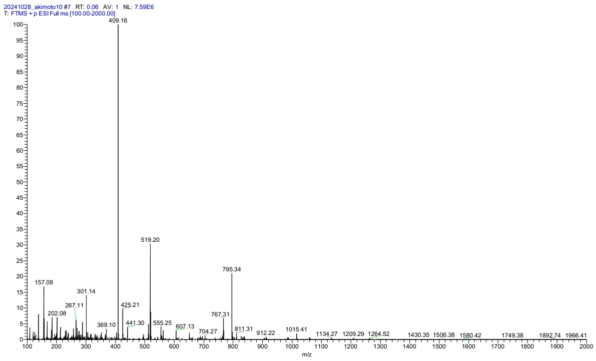

(19) Positive control (KA61 extract in strain KA20)

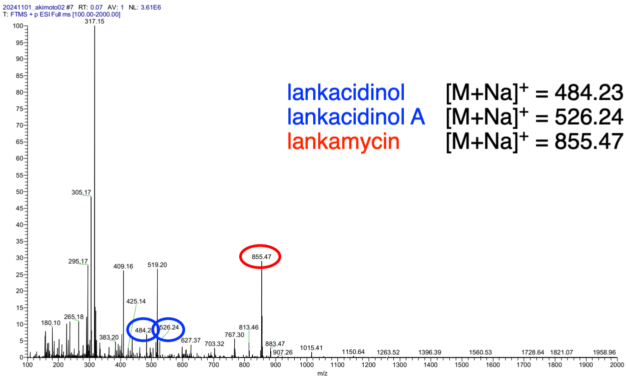

(20) Negative control (strain KA20)

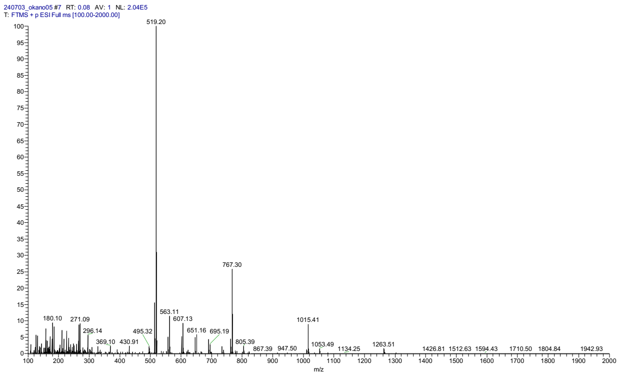

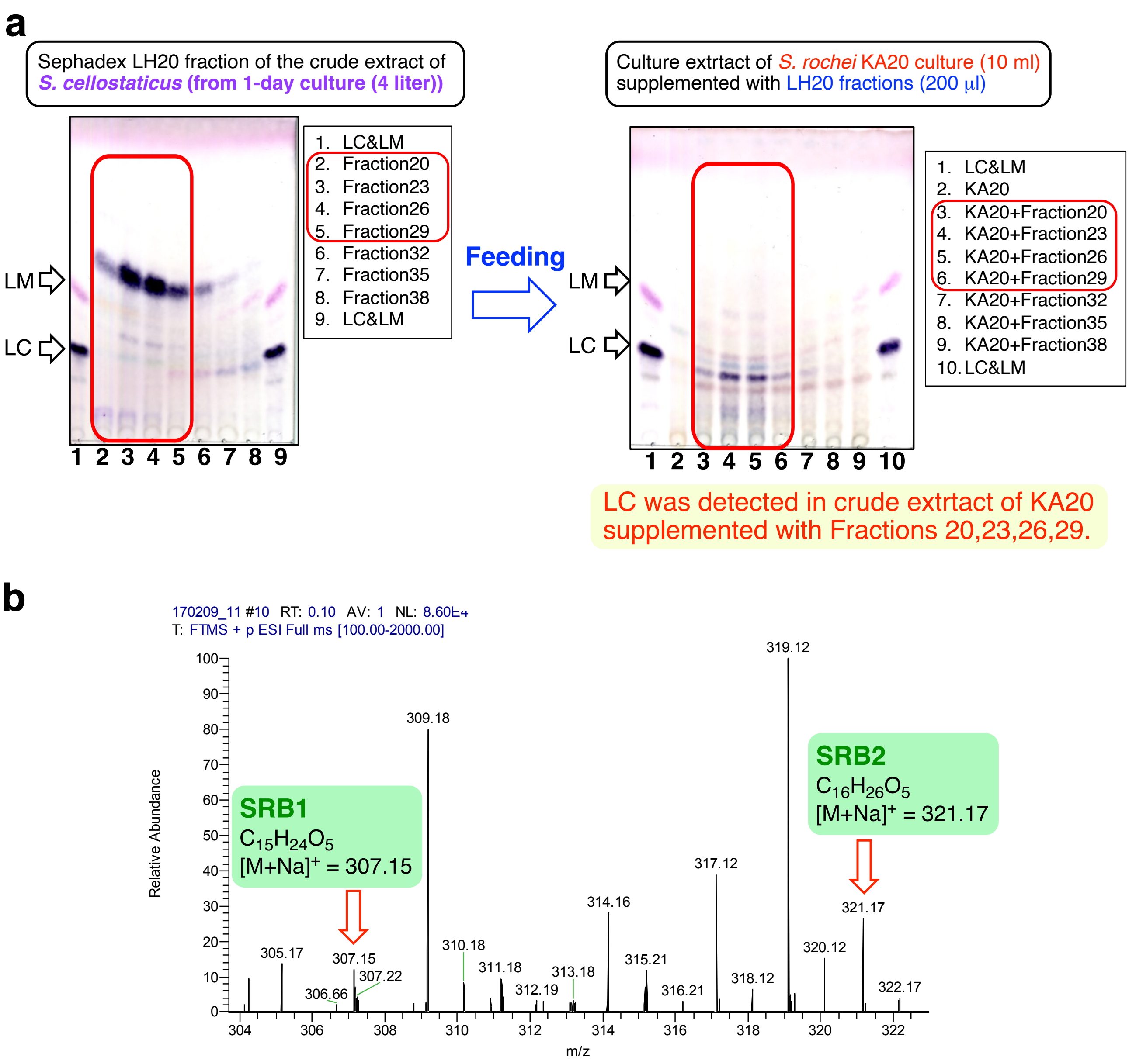

Figure S5. TLC and ESI-MS analyses of the fractions separated by Sephadex LH20 chromatography and complementation experiments using these fractions in *S. rochei* KA20. **A**) The crude extract of *S. cellostaticus* was partially purified by Sephadex LH20, and then these fractions were fed into *S. rochei* KA20. **B**) ESI-MS analysis of fraction 23 eluted from Sephadex LH20. The corresponding peaks for SRB1 and SRB2 were detected.

Figure S6. ESI-MS spectra of strains with higher antimicrobial activity when co-fermentated with *S. rochei* KA20 The followings are 8 culture strains exhibiting inhibitory zones when they were performed co-fermentation with *S. rochei* KA20 [(1) HUT6021, (2) HUT6072, (3) HUT6166, (4) HUT6228, (5) Tü113, (6) JCM5042, (7) NBRC13447, and (8) TOHO-M025]. Positive control [panel (9)] was also displayed. Detailed sample information was described in **Table S2**.

(1a) HUT6021 (+) (Co-fermentation with KA20)

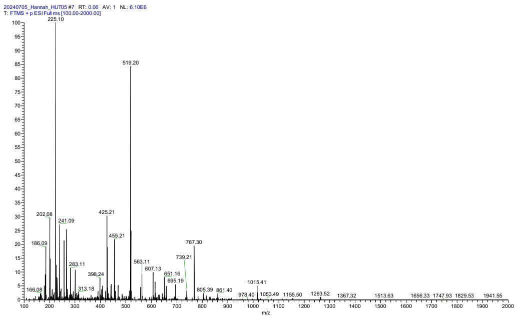

(1b) HUT6021 (–) (Single cultivation)

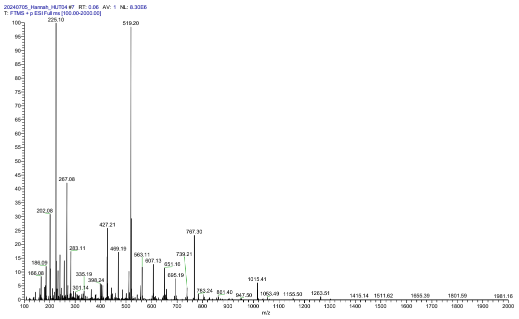

(2a) HUT6072 (+) (Co-fermentation with KA20)

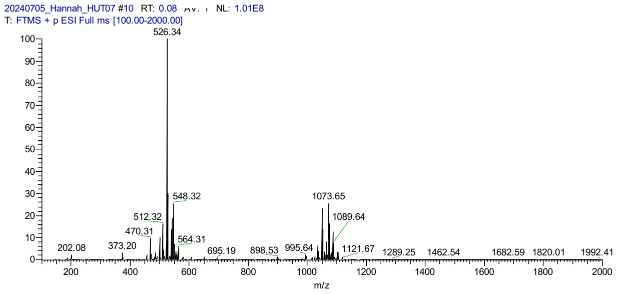

(2b) HUT6072 (–) (Single cultivation)

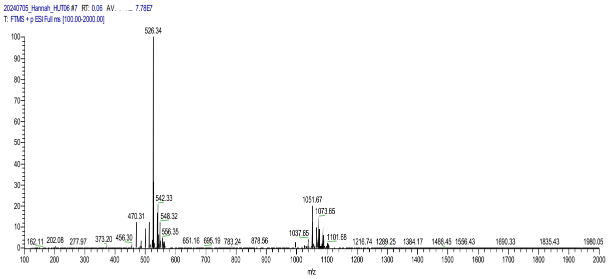

Figure S6. Continued.

(3a) HUT6166 (+) (Co-fermentation with KA20)

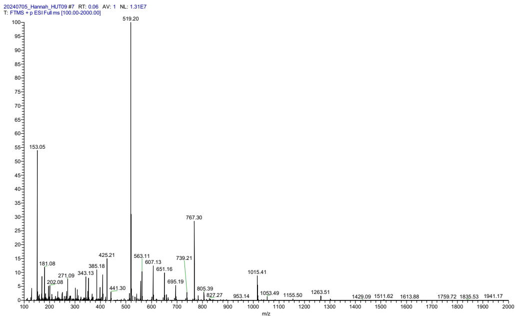

(3b) HUT6166 (–) (Single cultivation)

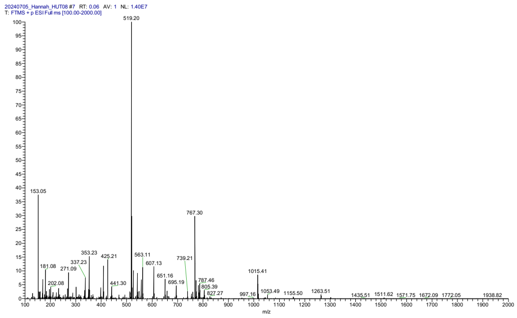

(4a) HUT6228 (+) (Co-fermentation with KA20)

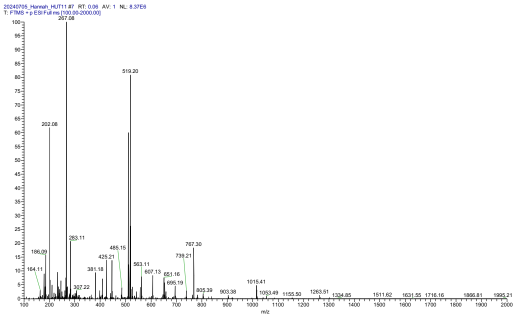

(4b) HUT6228 (–) (Single cultivation)

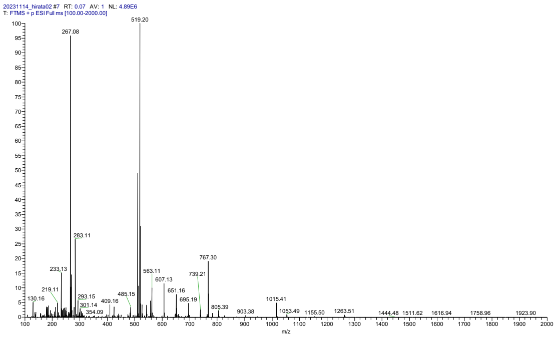

(5a) Tü113 (+) (Co-fermentation with KA20)

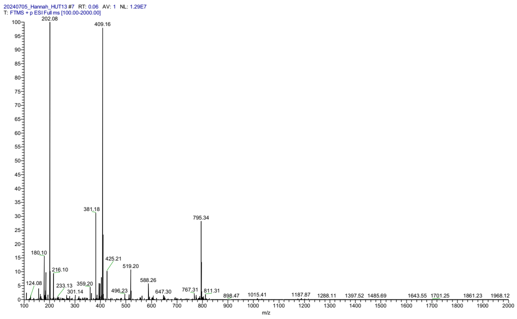

(5b) Tü113 (–) (Single cultivation)

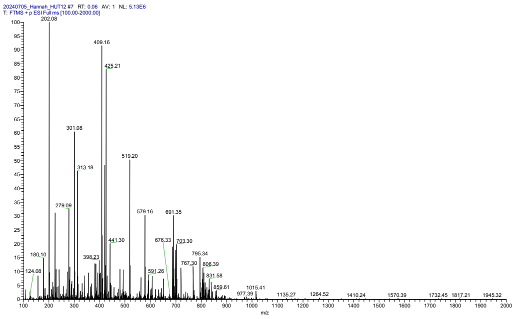

Figure S6. Continued.

(6a) JCM5042 (+) (Co-fermentation with KA20)

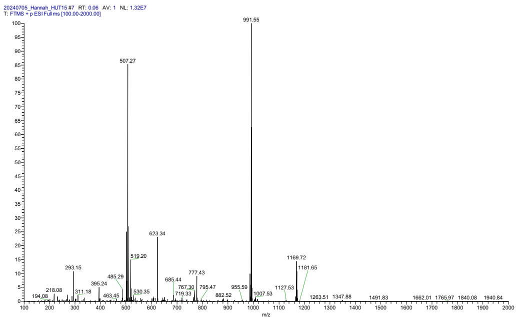

(6b) JCM5042 (–) (Single cultivation)

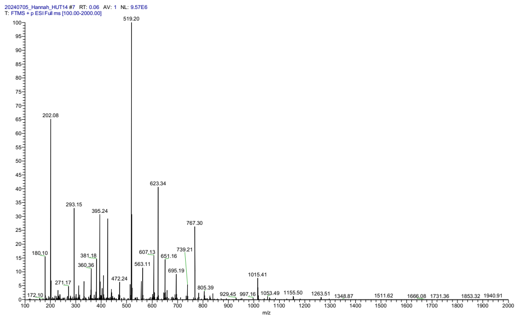

(7a) NBRC13447 (+) (Co-fermentation with KA20)

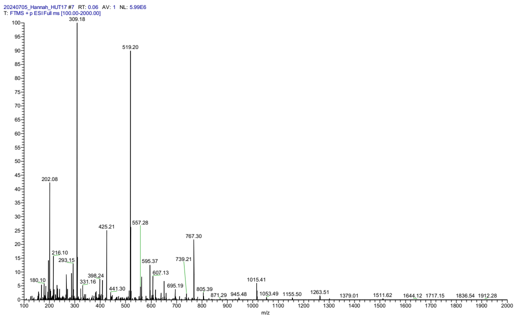

(7b) NBRC13447 (–) (Single cultivation)

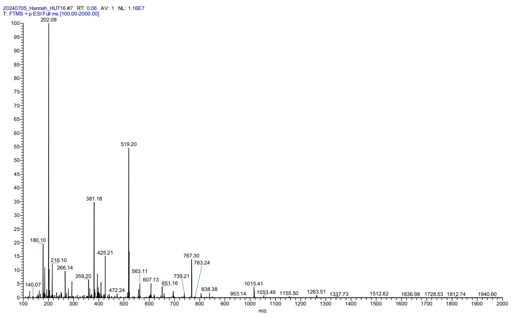

(8a) TOHO-M025 (+) (Co-fermentation with KA20)

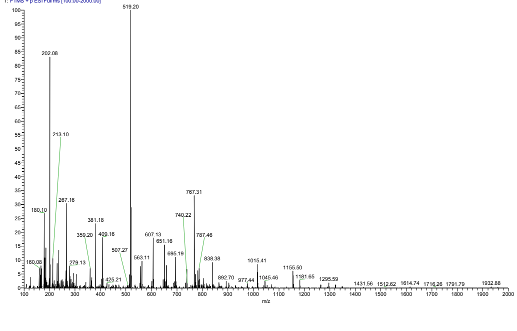

(8b) TOHO-M025 (–) (Single cultivation)

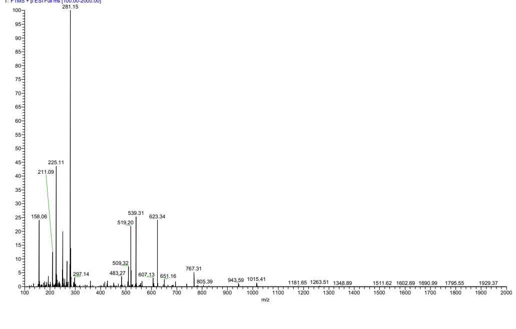

Figure S6. Continued.

(9a) KA61 (+) (Co-fermentation with KA20)

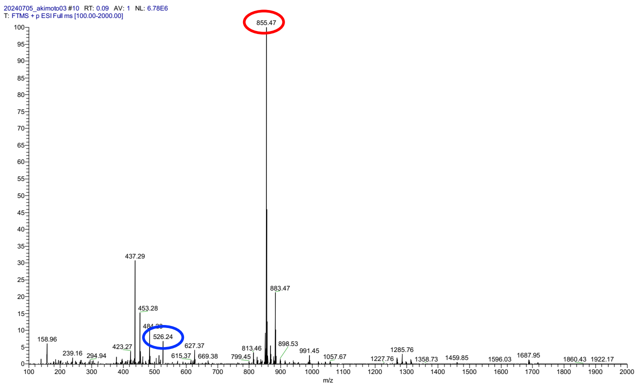

(9b) KA61 (–) (Single cultivation)

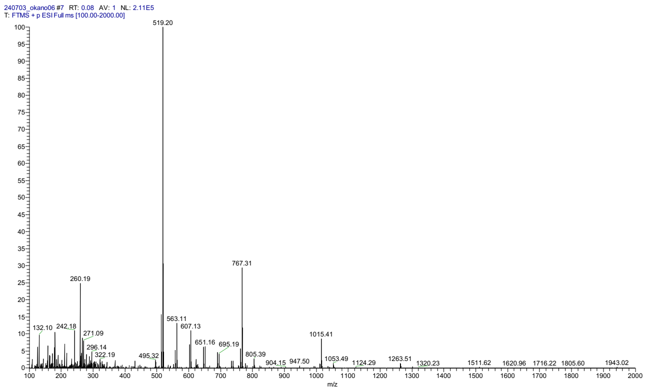

lankacidinol A $[M+Na]^+$ = 526.24

lankamycin $[M+Na]^+$ = 855.47

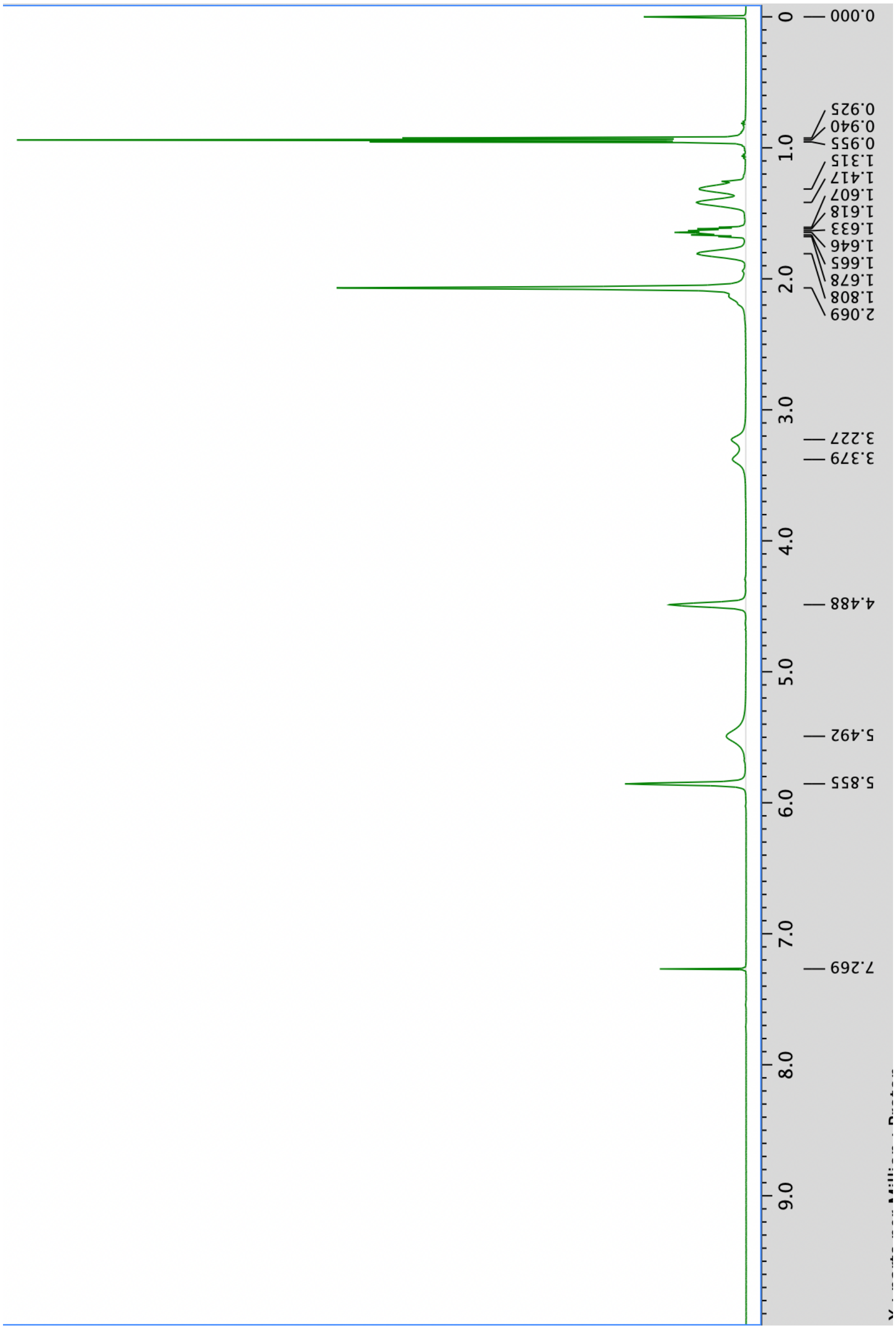

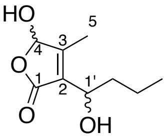

SAB1

Figure S7. ^{1}H-NMR spectrum of SAB1.

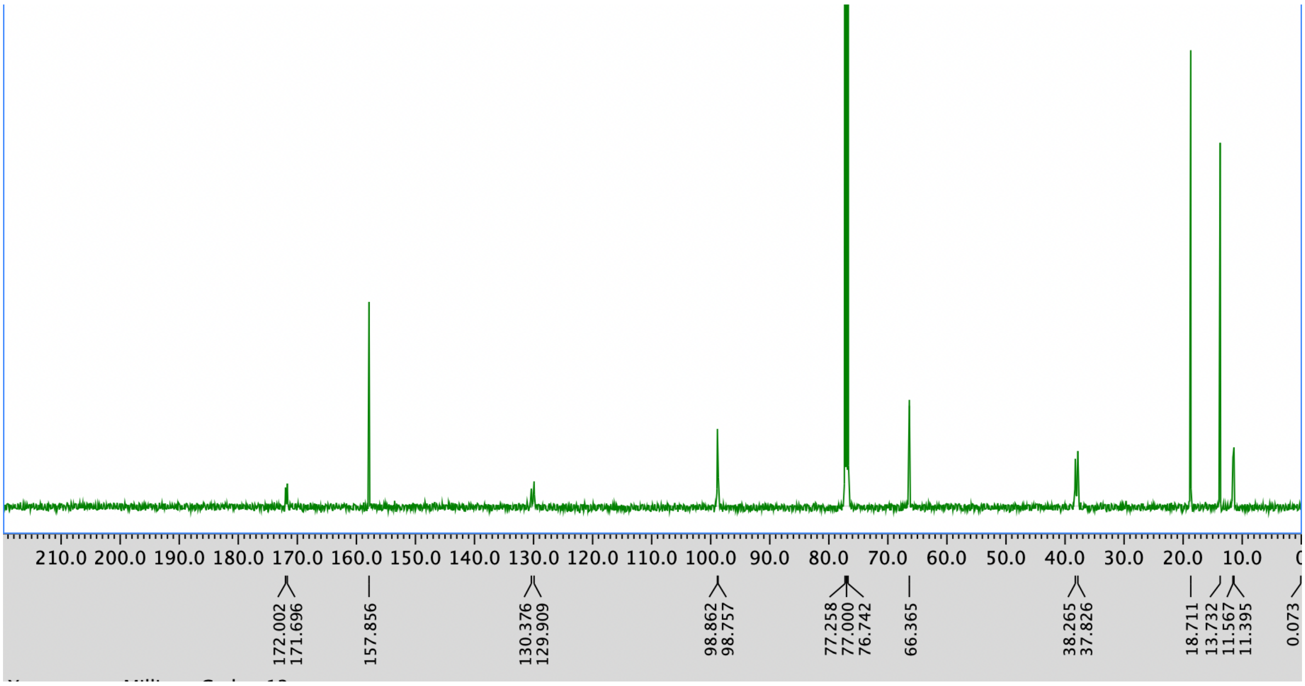

Figure S8. ^{13}C-NMR spectrum of SAB1.

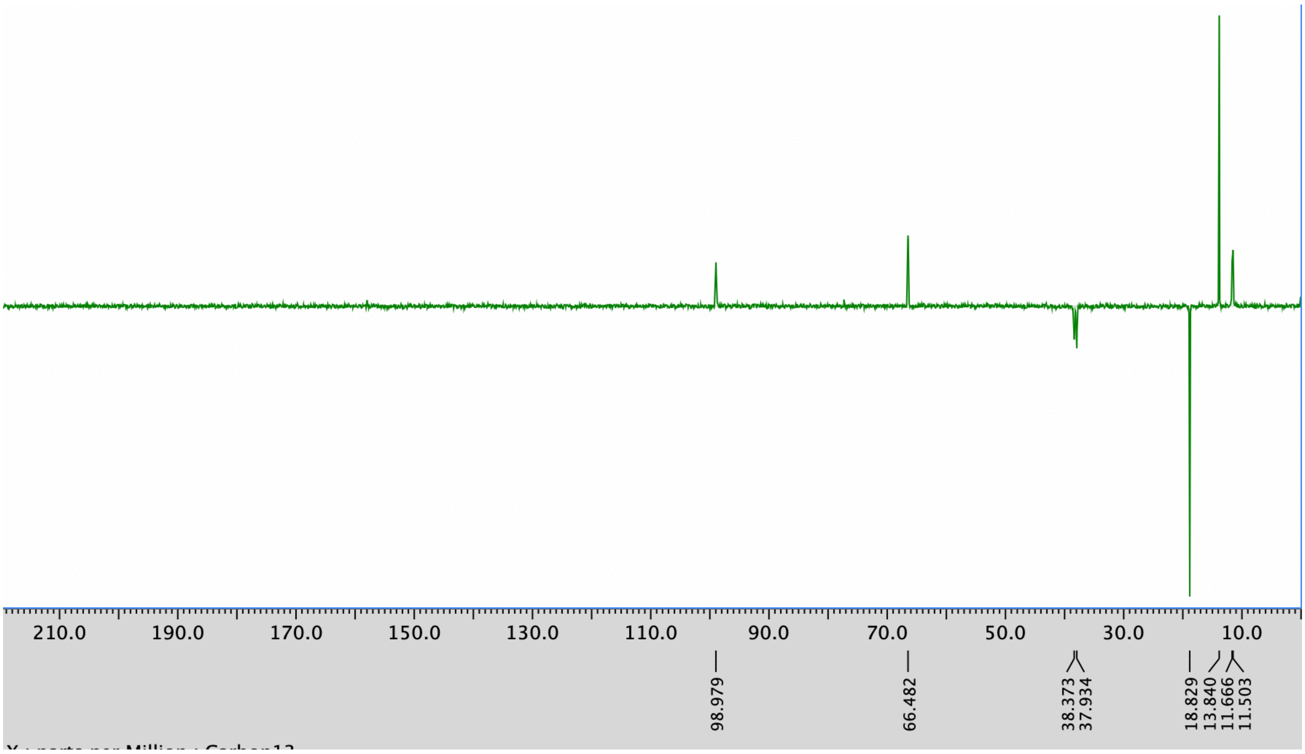

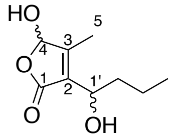

SAB1

Figure S9. DEPT135 spectrum of SAB1.

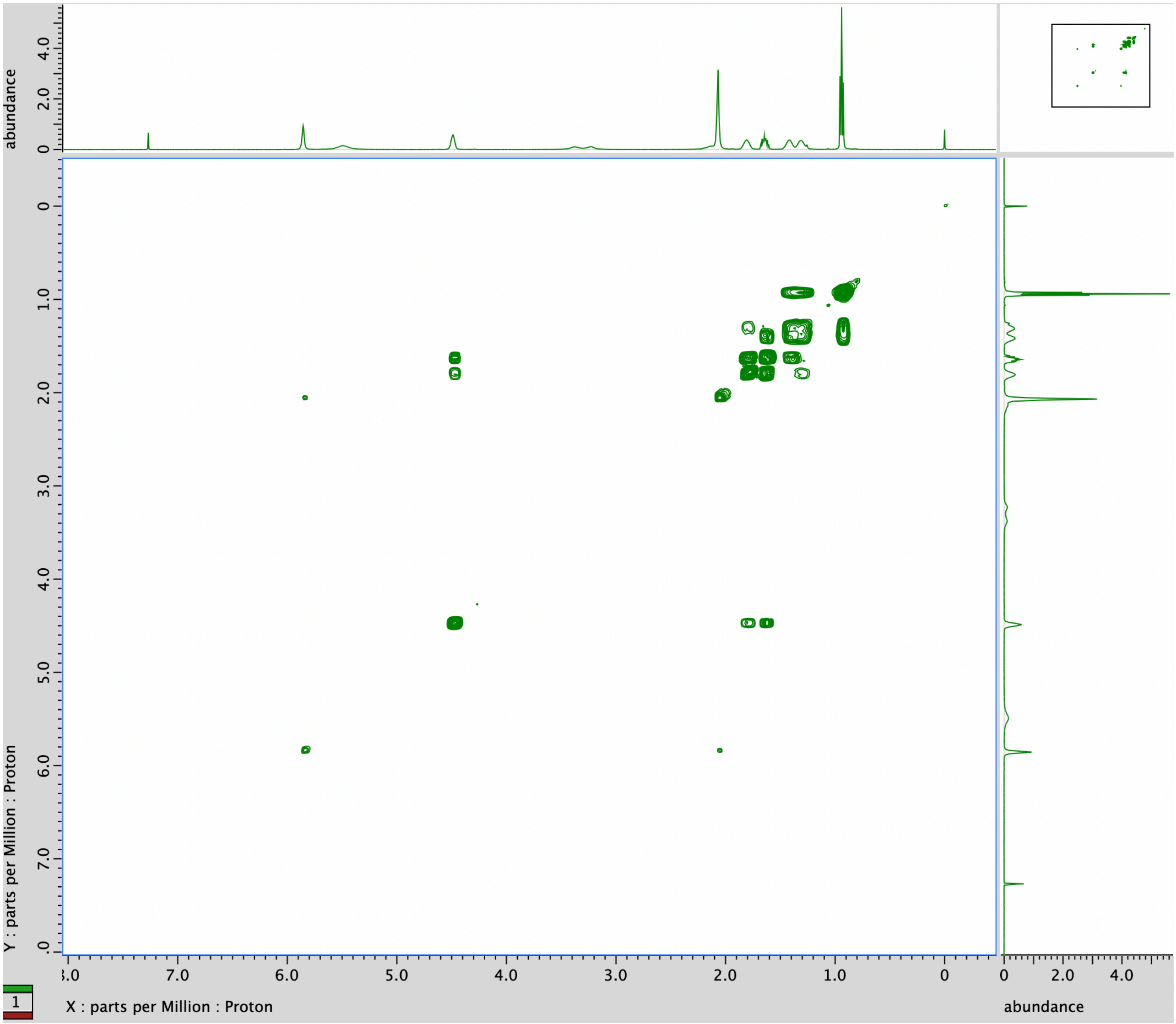

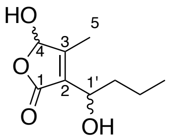

SAB1

Figure S10. DQF-COSY spectrum of SAB1.

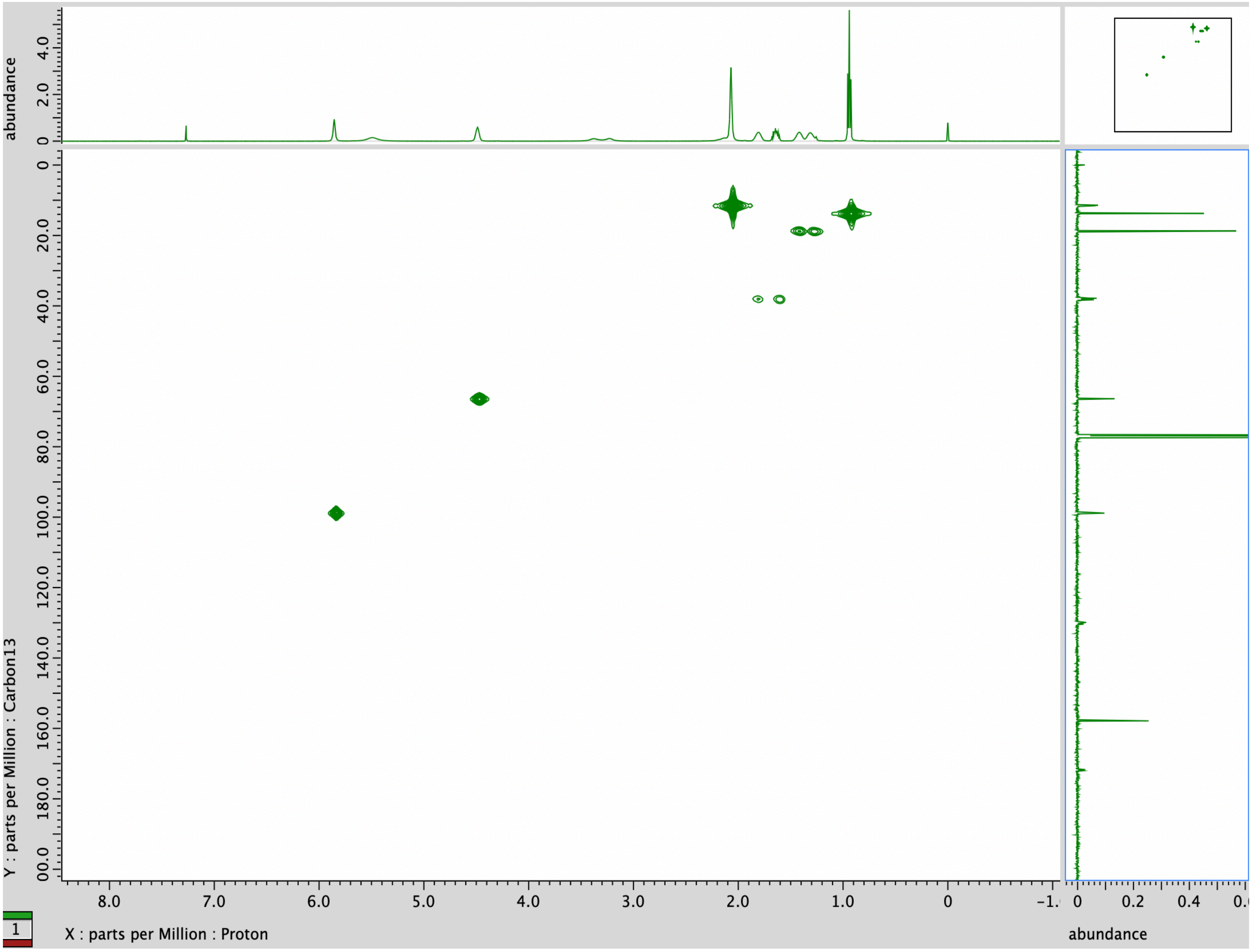

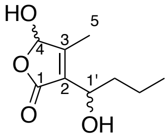

SAB1

Figure S11. HMQC spectrum of SAB1.

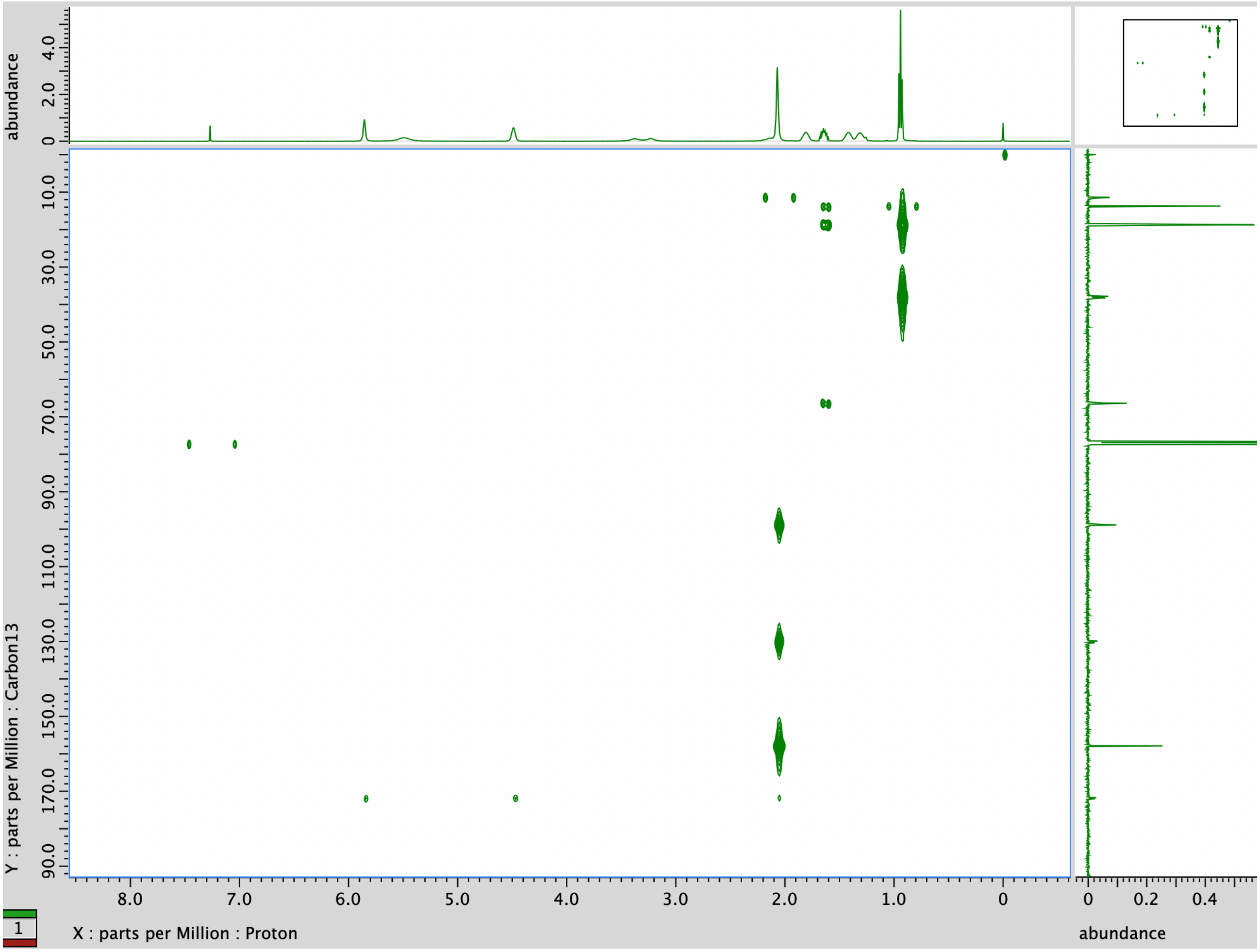

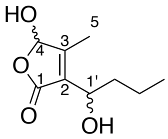

SAB1

Figure S12. HMBC spectrum of SAB1.

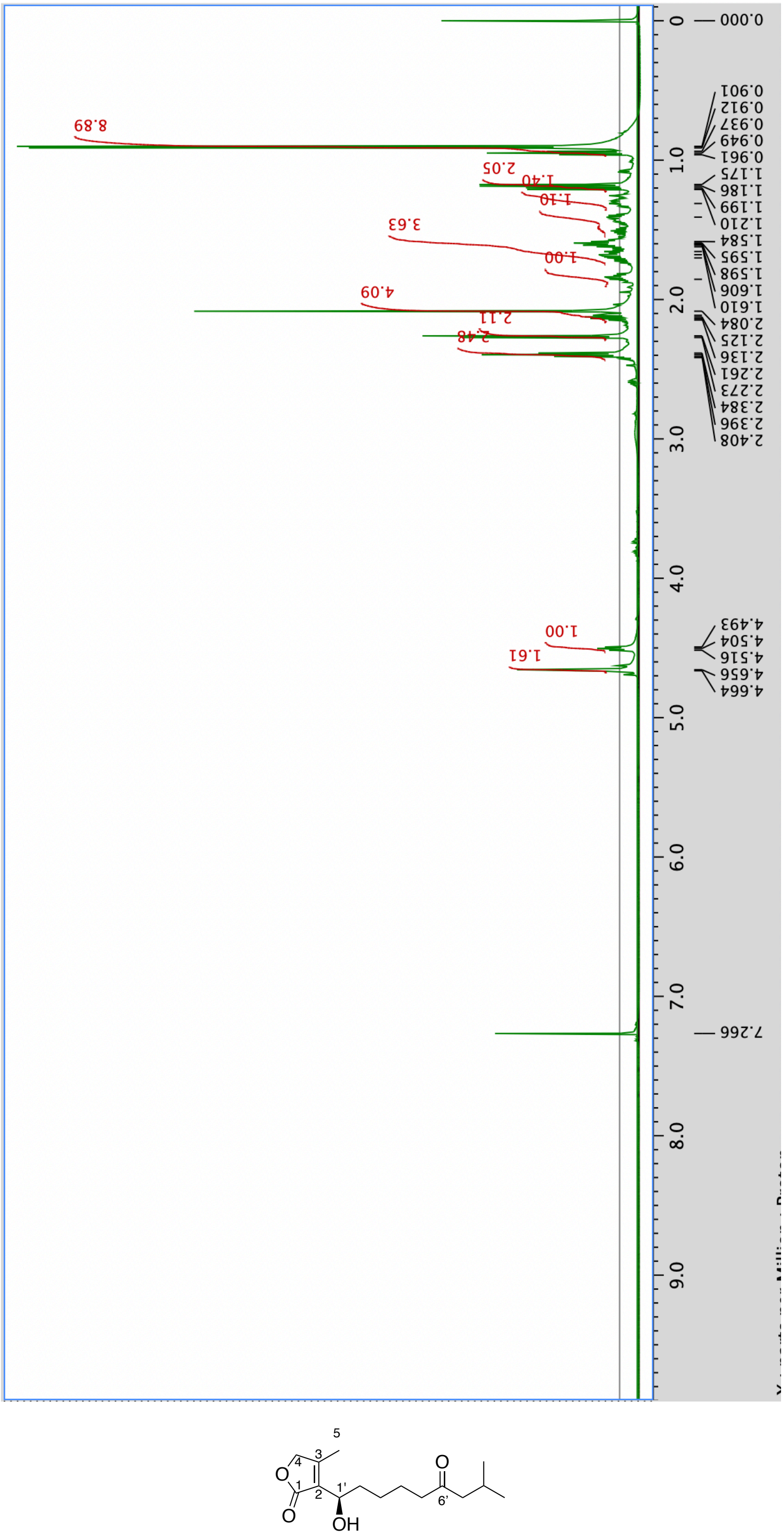

4-Dehydroxy-SRB1

Figure S13. ^{1}H-NMR spectrum of 4-dehydroxy-SRB1.

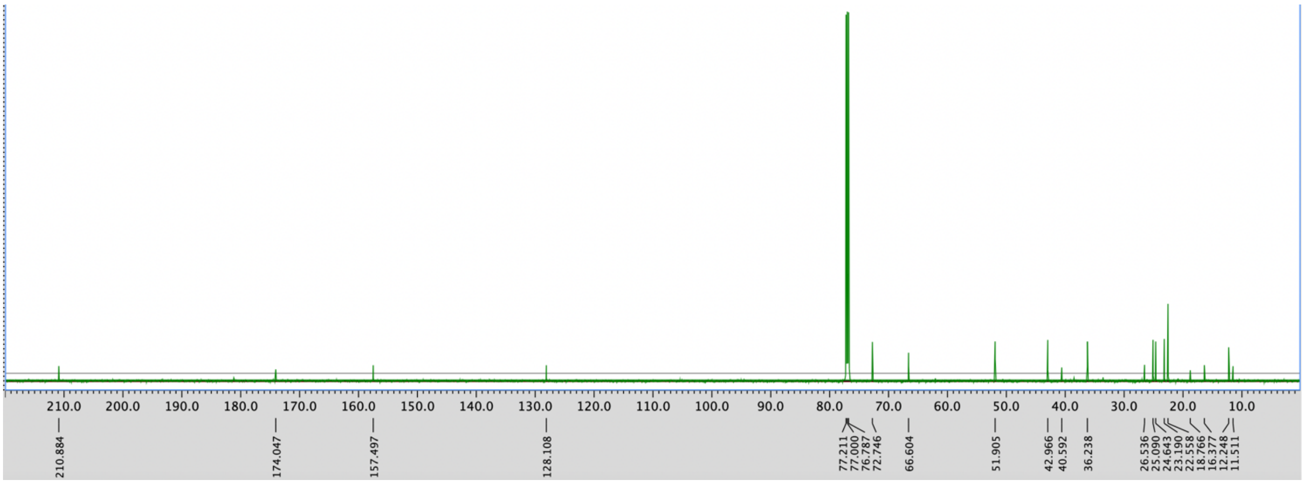

Figure S14. ^{13}C-NMR spectrum of 4-dehydroxy-SRB1.

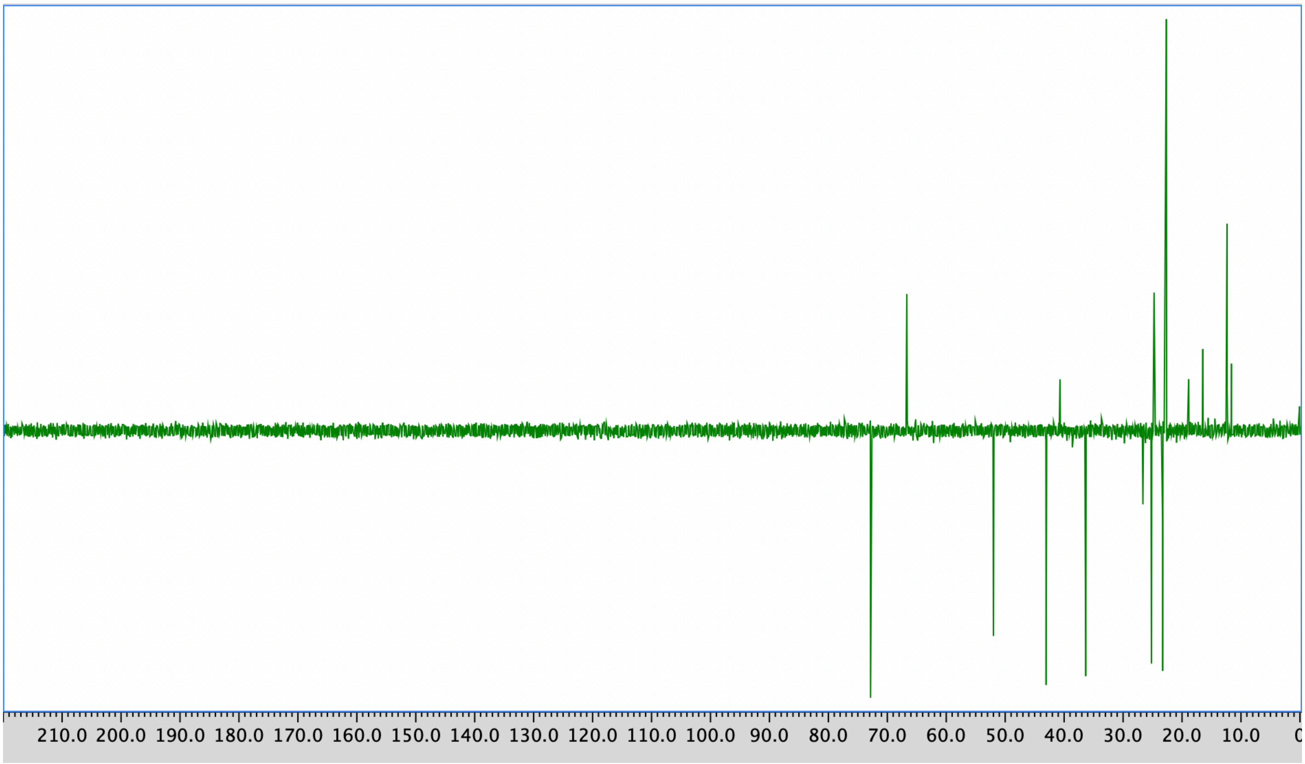

Figure S15. DEPT135 spectrum of 4-dehydroxy-SRB1.

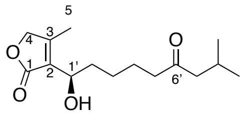

4-Dehydroxy-SRB1

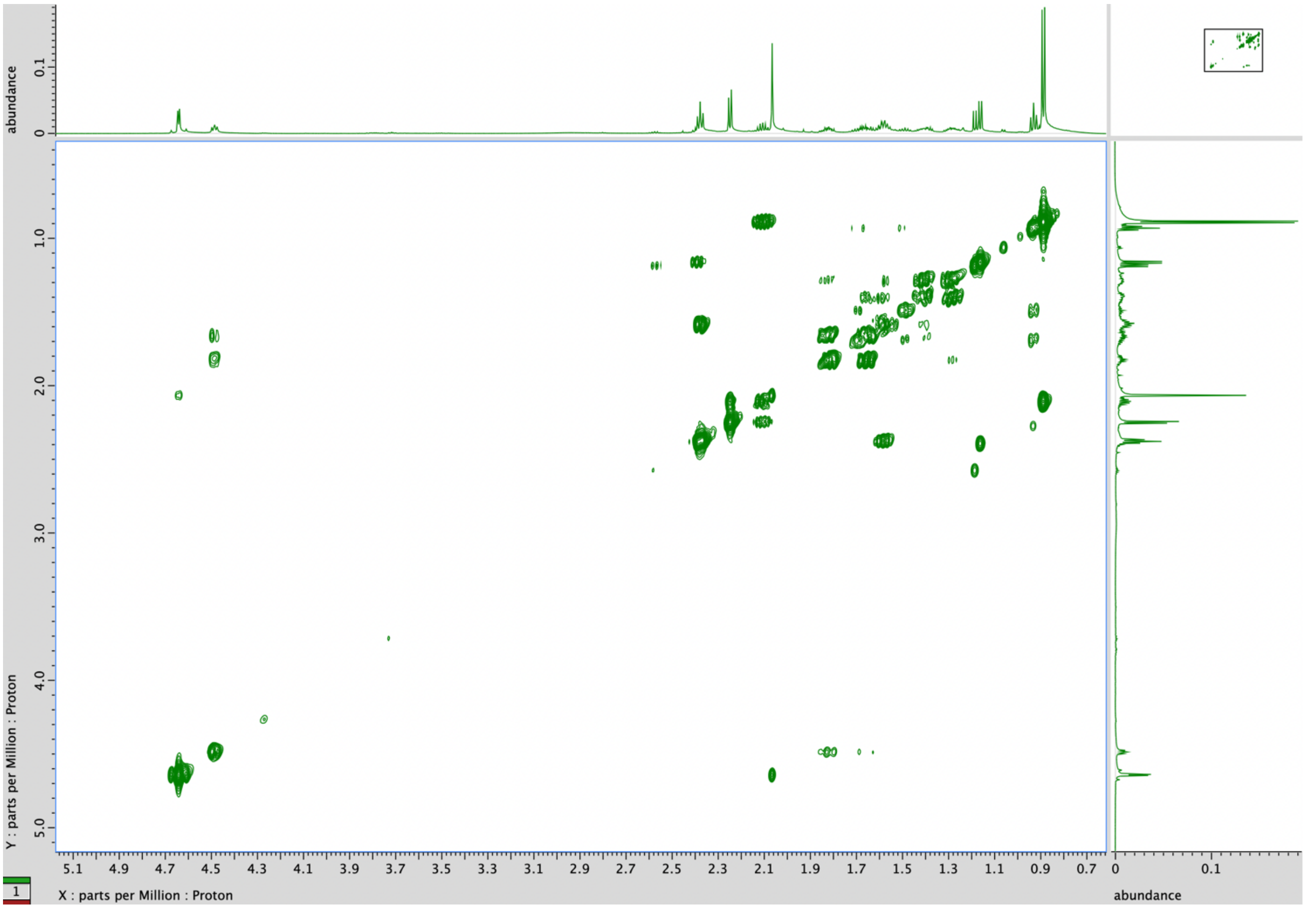

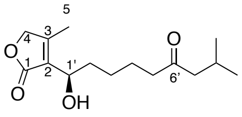

4-Dehydroxy-SRB1

Figure S16. DQF-COSY spectrum of 4-dehydroxy-SRB1.

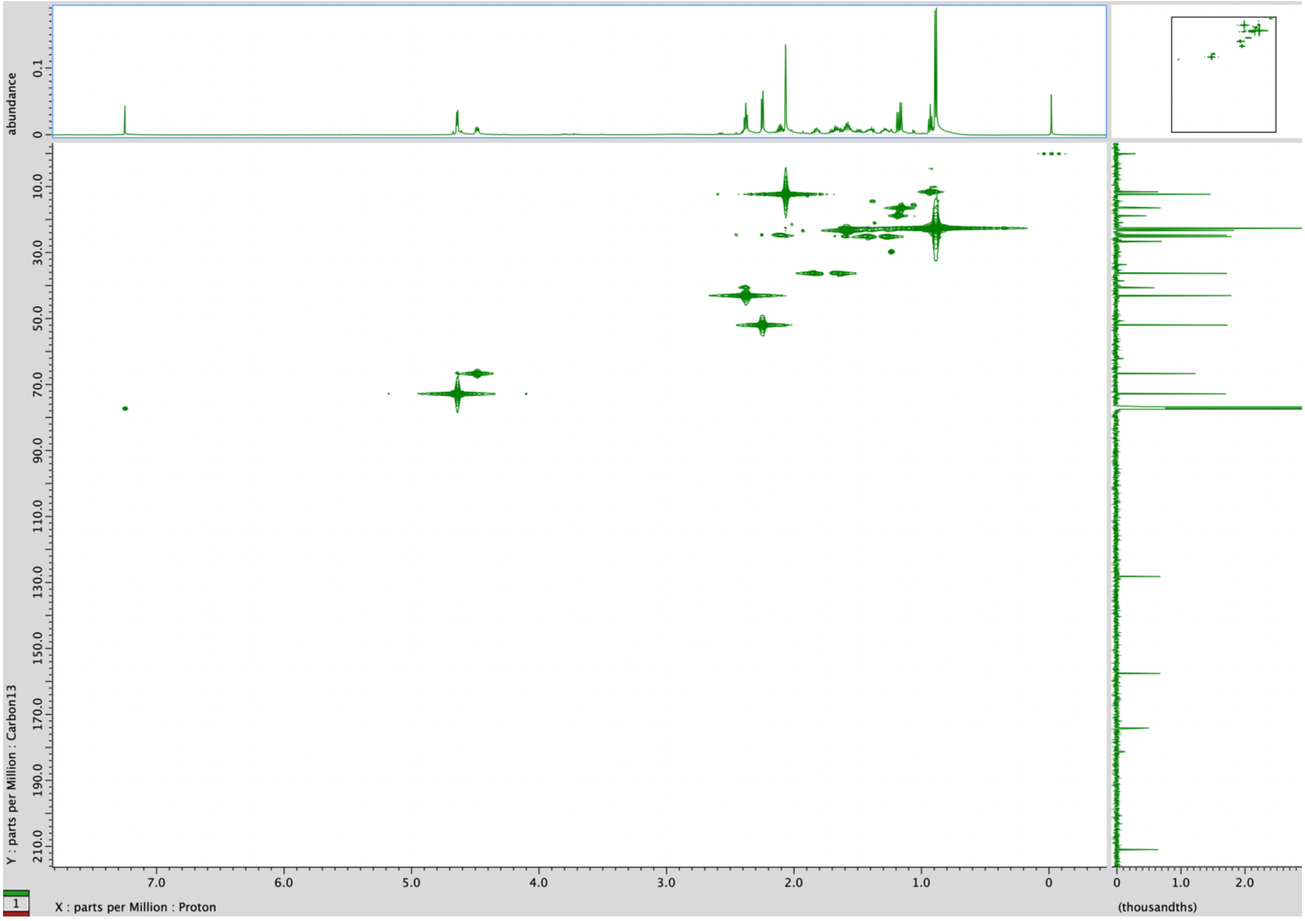

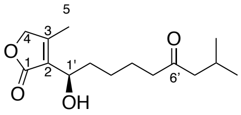

4-Dehydroxy-SRB1

Figure S17. HMQC spectrum of 4-dehydroxy-SRB1.

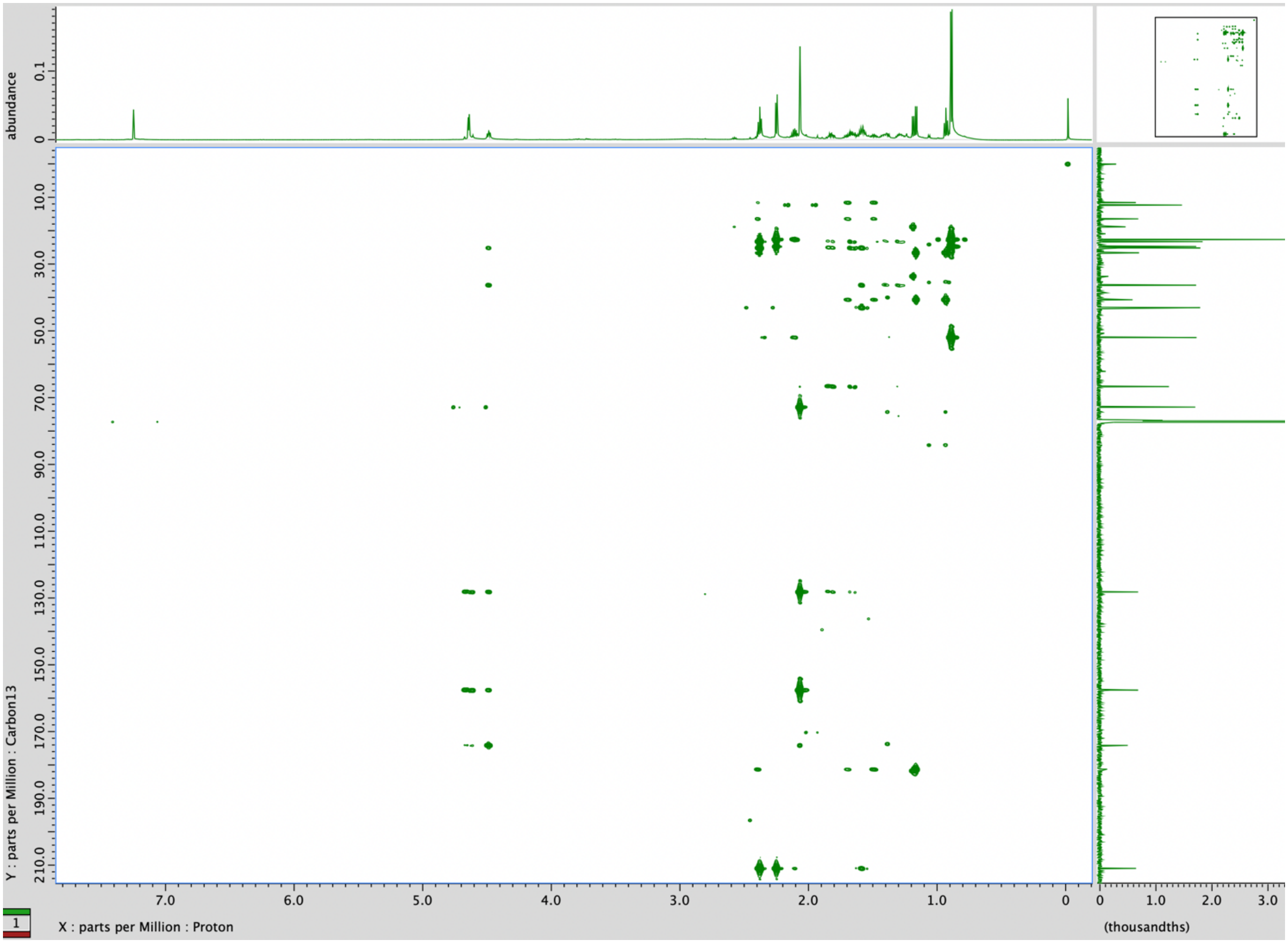

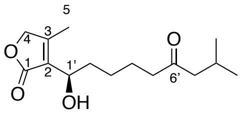

4-Dehydroxy-SRB1

Figure S18. HMBC spectrum of 4-dehydroxy-SRB1.
